# Nanoprecipitation and Drug Delivery with PMTC: Toward Biomedical Application of Polyesters from Radical Ring‐Opening Polymerization

**DOI:** 10.1002/mabi.202500432

**Published:** 2025-10-19

**Authors:** Eleni Axioti, Fabian Mehner, Morgan Reynolds‐Green, Aniket R. Bukane, Robert J. Cavanagh, Stefan Michel, Günter K. Auernhammer, Vincenzo Taresco, Jens Gaitzsch

**Affiliations:** ^1^ School of Chemistry University of Nottingham Nottingham UK; ^2^ Leibniz‐Institut für Polymerforschung Dresden e.V. Dresden Germany; ^3^ Technische Universität Dresden Faculty of Chemistry and Food Chemistry Organic Chemistry of Polymers Dresden Germany; ^4^ School of Pharmacy University of Nottingham Nottingham UK

**Keywords:** drug delivery, nanoparticles, nanoprecipitation, polyester, RROP

## Abstract

Polymerized cyclic ketene acetals (PCKAs) prepared by radical ring‐opening polymerization (RROP) have shown tremendous potential in the biomedical field. In this work, the field is expanded to the formation of fully degradable nanoparticles (NPs) from the fast‐degrading poly(2‐methylene‐1,3,6‐trioxocane) (PMTC). The formulation of homopolymers is typically challenging due to their lack of amphiphilicity; however, implementing nanoprecipitation as a robust, fast, and cost‐efficient method of self‐assembly has yielded well‐defined polymeric nanocarriers of 100–200 nm in diameter. The characterization of hydrophilicity and dye‐encapsulation mediated via different polyester degrees of branching has enabled insights into utilizing this key characteristic of RROP. The degree of branching affected dye encapsulation in the absence of altering hydrophilicity. The highest levels of encapsulated Coumarine‐6 (Cou6) as a model drug were found with polymers possessing an intermediate degree of branching (8%) at low molecular weight (9 kg/mol). In addition to stable NPs, the disassembly of these NPs in extreme pH regions promised potential for targeted drug delivery. In vitro studies have demonstrated the cytocompatibility of NPs and their degradation products, and their ability to achieve cell uptake of Cou6‐loaded NPs has been confirmed, highlighting the potential of PMTC‐NPs as drug delivery vehicles. The successful protocol to prepare NPs purely of polymerized CKAs demonstrated here thus enables future efforts to expand the library of NPs from RROP.

## Graphical abstract

Homopolyester from Radical Ring‐Opening‐Polymerisation (RROP) have been applied in nanoprecipitation to form nanoparticles for drug delivery. These nanoparticles are stable over various weeks and showed no toxicity. Encapsulated Coumarine has been delivered to cells to show the full potential of these biodegradable delivery systems from RROP.

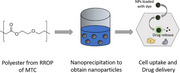

## 1 Introduction

The physical stability and chemical versatility of polymeric nanoparticles (NPs) have made them attractive candidates in the biomedical field for promoting targeted drug delivery and continuous drug release [1, 2]. Of particular interest are biodegradable, biocompatible polymers, either as amphiphilic block‐copolymers or homopolymers, that can form NPs and mediate controlled drug release upon polymer degradation [3, 4].

A promising technique to prepare biodegradable polymers is the radical ring‐opening polymerization (RROP) of cyclic ketene acetals (CKAs) [5, 6, 7, 8, 9]. Whilst not all polymerized CKAs (PCKAs) have been fully characterized, a few have shown biocompatibility in addition to their biodegradation [10, 11, 12, 13]. Combining the synthetic flexibility of a radical polymerization and addressing the issue of missing degradability of common materials in biomedicine, Nicolas et al. showcased the potential of nanoparticles included from RROP‐based components [14, 15]. Starting with a PEG‐ or vinylene‐based macroinitiator and propagating with CKAs, partially degradable block copolymers were prepared, which could be successfully assembled into NPs in the next step [9, 14, 16, 17, 18, 19, 20, 21]. In a drop‐in approach, partially degradable block copolymers, statistical and gradient copolymers of vinylenes and CKAs were prepared, allowing for a plethora of functional, degradable materials. Tuning the hydrophilicity of the respective vinylic‐ or CKA‐component allowed tuning the degradation rate of the NPs [4, 9, 14, 15, 16, 18, 19, 20, 21, 22, 23, 24]. Moving from the hydrophobic 4,5‐benzyl‐2‐methylenedioxepane (BMDO) to 2‐methylenedioxepane (MDO) and the least hydrophobic CKA, 2‐methylene‐1,3,6‐trioxocane (MTC), as comonomers, the fastest degradation into the residual vinylic oligomers was observed for copolymers with MTC [6, 25, 26].

These works already outline the potential of PCKA‐containing nanoparticles, indicating the potential for fully degradable nanoparticles from PCKA homopolymers. In recent years, a variety of publications showcased new CKAs and their polymers, including responsive polyesters from amine‐bearing CKAs copolymerized with MTC, sugar‐based CKAs derived from renewable resources, and redox‐functionalities resulting from thioester‐functions, further motivating to use of PCKA‐homopolymers without additional vinylic components [5, 6, 25, 26, 27, 28]. Block‐copolymers of PEG‐PMTC have hinted that PMTC is a strong candidate for drug release [21]. The aim of the current work is to develop a formulation strategy for PMTC to enable its potential in biomedical applications.

Whilst formulation strategies differ from homo‐ to block‐copolymers, in all approaches a high control in the shape and size of the prepared NPs is desired. Well‐defined amphiphilic block‐copolymers are assembled by a phase separation between the hydrophilic and hydrophobic blocks. Various particle shapes and sizes can be generated; however, low‐disperse, well‐defined polymers are required [29, 30, 31, 32, 33]. Considering the difficulty in achieving low dispersities in polycondensation or RROP, this formulation strategy is only suitable for polymers from controlled polymerization techniques. To assemble less defined block copolymers with broad dispersities, or homopolymers with missing amphiphilicity, emulsification or nanoprecipitation techniques were developed [32, 34]. A drawback of conventional emulsification is the need for energy‐intensive protocols involving high‐shear force (e.g., ultrasonication) or the addition of potentially harmful stabilizing surfactants [35].

**FIGURE 1:**
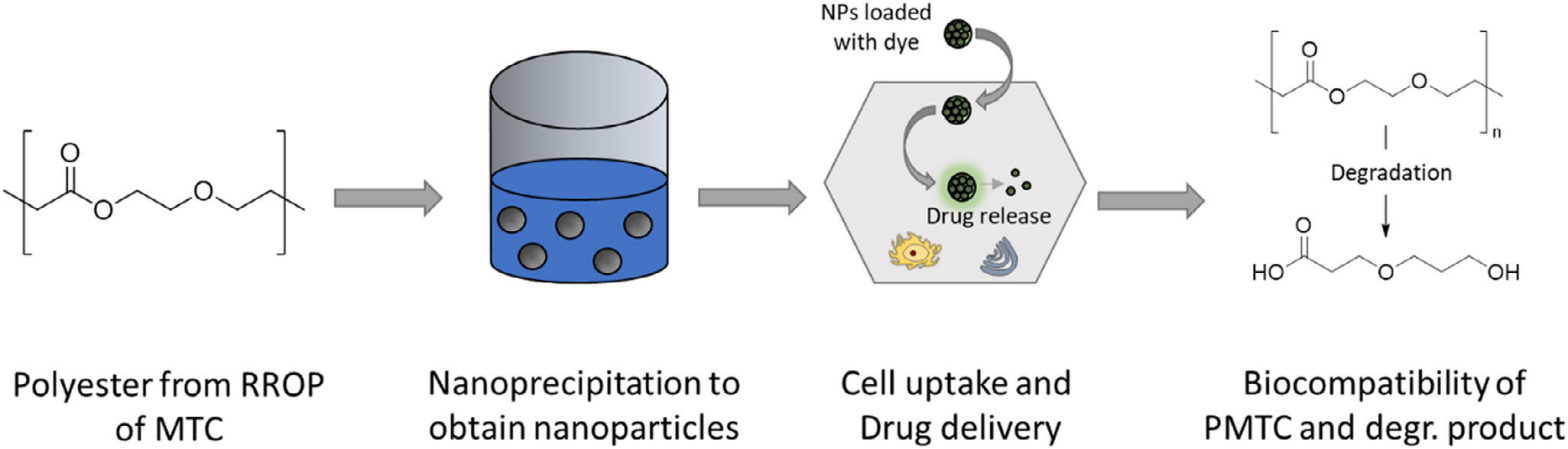
Schematic representation of this work, starting with the structure of PMTC that was used to form nanoparticles via nanoprecipitation and loaded with a dye to be taken up by cells as a test for drug delivery. Both the initial polymer and the degradation products are tested for their biocompatibility.

Overcoming these issues, nanoprecipitation protocols as a one‐step, low‐energy, and low‐cost protocol were developed and allowed to prepare spherical nanoparticles [35, 36, 37]. By simply dissolving the polymer in a volatile solvent such as THF and dropwise addition to deionized water, polymer nanoparticles can be prepared [37, 38, 39, 40]. The rapid formation of nanoparticles is driven by the Marangoni effect, which is caused by interfacial turbulence between the solvent (e.g., THF) and non‐solvent (water) [41]. In numerous reports, kinetically stable NPs with tunable dimensions could be prepared with this method for glycerol‐ and diglycerol‐based polyesters, or poly(ε‐caprolactone)‐based block‐copolymers without the addition of surfactants (Figure 1) [35, 42, 43, 44, 45]. Simple coprecipitation of dyes and drugs then enabled the encapsulation of these compounds [37, 42]. This concept has inspired the application of PCKA homopolymers in nanoprecipitation toward drug‐loaded nanoparticles for biomedicine.

This work bridges the advantages of fully degradable RROP homopolymers with the advantages of nanoprecipitation as a fast and easily applicable method to form NPs. PMTC has been reported to be the fastest degrading PCKA and is used to develop NPs as a drug delivery system (Figure 1) [13, 21]. Due to missing crystallinity and high chain flexibility due to its internal PEG‐analogue ether‐function, it was chosen as an ideal candidate to test the formulation potential of PCKA‐homopolymers [28]. The current work investigates the correlation between polymer branching density and key particle characteristics, including PMTC hydrophobicity, solubility, particle size, particle stability, and drug‐encapsulation. Moreover, to study the biomedical application of the generated particles, cytotoxicity and particle uptake have been assessed in vitro.

## 2 Materials and Methods

### 2.1 Materials

Phosphate buffer saline (PBS), fetal bovine serum (FBS), chloroacetaldehyde dimethyl acetal, DOWEX WX2, *tert*‐butanol, potassium *tert*‐butoxide, AIBN, citric acid, sodium phosphate dibasic, lipase from porcine pancreas (Type II, ≥ 125 units/mg), and coumarin 6 were purchased from Sigma–Aldrich. Tetrahydrofuran and dimethyl ether were purchased from Fisher Scientific UK. All chemicals and solvents were used as received without further purification.

### 2.2 Methods

#### 2.2.1 Synthetic Procedures

##### 2.2.1.1 2‐Chloromethyl‐1,3,6‐trioxocane (MTC‐Cl) and 2‐methylene‐1,3,6‐trioxocane (MTC)

The synthesis of MTC and MTC‐Cl was performed as reported in the literature and is described in detail in Section S1 [46]. Briefly, diethylene glycol, chloroacetaldehyde dimethyl acetal, and DOWEX WX2 were mixed, heated up to 120°C, and stirred for 3 h. The product was distilled to obtain MTC‐Cl as a white crystalline powder. The MTC‐Cl was then dissolved in *tert*‐butanol, before potassium *tert*‐butoxide was slowly added. The mixture was then heated to 120°C and stirred overnight. The product was extracted with diethyl ether and distilled multiple times to provide MTC as a clear, transparent liquid.

##### 2.2.1.2 Polymerization of MTC

The synthesis of PMTC was followed by the thermal polymerization reported in literature [46]. In brief, MTC and AIBN were mixed and degassed with a gentle argon flow for 15 min. The mixture was then stirred at 85°C and quenched by cooling the mixture with ice and opening the reaction vessel. The product was worked up by precipitation in ice‐cold diethyl ether, and the precipitate was dried in a vacuum oven at 50°C. As discussed in a previous work, the density of branches (DB) and molar mass of PMTC prepared by RROP were solely dependent on the conversion and independent of the reaction conditions [46]. For that reason, the polymers with a low molar mass had a limited DB below 10% and the high molar mass samples could not be prepared with a low DB. The sample names discussed in this manuscript follow the molar mass and DB as follows: PMTC‐M_n_‐DB. PMTC‐7‐9 has a molar mass of 7 kg/mol and a DB of 9%. For detailed synthesis procedures and the exact analysis data, see Table S1.

##### 2.2.1.3 Preparation of the Hydrolysis Product

In order to test the biocompatibility of the degradation product of PMTC, the polyester was degraded under alkaline, accelerated conditions. Following the protocol from Nicolas et al., 0.23 g of PMTC was dissolved in 9.2 mL THF [9]. A mixture of 0.5 g NaOH in 4.8 mL Methanol was added, and the reaction mixture was stirred at 25°C. The previous colorless reaction mixture turned turbid when the degradation products were formed. After 24 h, the reaction mixture was neutralized by slowly adding H_2_SO_4_. The sample was dried overnight in a vacuum drying oven and analyzed by ^1^H NMR (Figure S1). The degradation product was then used for the biocompatibility tests.

#### 2.2.2 Characterization

##### 2.2.2.1 Size Exclusion Chromatography (SEC)

To analyze the samples for their molar mass, a similar protocol to previous studies was used [46, 47]. SEC, coupled with a triple detection setup, was used and comprised of a degasser, an isocratic pump (both series Agilent 1200), an autosampler (Agilent series 1100), and 2 x SEC columns PLgel MIXED‐C columns (300–7.5 mm;5 µm particle size) by Agilent Technologies Inc. (USA). The downstream detection system was comprised of a multi‐angle light scattering MALS DAWN HELEOS II, a viscometric detector Viscostar III, and a differential refractometer Optilab T‐rEX, all by Wyatt Technology Corp. (USA). Separations were performed in THF (stabilized with 0.025% BHT) as eluent with a flow rate of 1 mL/min. A polymer solution (53 µL) was injected at an analyte concentration of 4 mg/mL. Data recording and analysis were performed using the software Astra by Wyatt Technology Corp. (USA), version 7.3.2. Initial MALS detector normalization, delay volume alignment, and band broadening correction were adjusted by measuring a narrowly distributed PS standard with a M_w_ of 30 000 g/mol (PS80317, Pressure Chemicals Co, USA) and Đ ≤ 1.06, validated by measurements of other PS standards with higher M_w_ (200 000 g/mol).

##### 2.2.2.2 NMR Spectroscopy

NMR measurements were performed using a Bruker Advance III 500 spectrometer operating at 500.13 MHz for ^1^H and at 125.74 MHz for ^13^C. Deuterated chloroform (CDCl_3_) was used as solvent and reference (δ(^1^H) = 7.26 ppm; δ(^13^C) = 77.0 ppm). The measurements were carried out at 30°C using standard pulse sequences of the TopSpin 3.2 software package (Bruker Biospin). Following the protocol discussed in the literature, a deviation of 5% was implemented for the determination of the DB [46].

##### 2.2.2.3 Solubility Tests

For the solubility tests, 25 mg of PMTC were dissolved in 10 mL THF. With a PerkinElmer Lambda 800 UV/Vis Spectrophotometer, the transmission at 800 nm was measured. Water was slowly added, first in 1 mL steps, then in 0.2 mL steps, and the transmission of the solution was measured. After reaching the cloud point, the polymer precipitated, and transmittance decreased. From the onset, where the transmittance decreased, the turbidity point was determined.

##### 2.2.2.4 Spin‐Coating and Contact Angle Measurement

For contact angle measurements, a thin film of PMTC on a Si‐wafer was prepared. For this, a piece of a wafer was cleaned by ultrasonication in acetone for 15 min, followed by another 15 min of ultrasonification in isopropanol. The wafer was then rinsed with isopropanol and dried with nitrogen gas. The cleaned wafer was then spin‐coated with a solution of 20 mg/mL PMTC in chloroform at a spinning rate of 5000 rpm. The solvent was removed in a drying oven at 40°C, and the film was analyzed by contact angle measurement using OCA 35L from DataPhysics Instruments GmbH, Filderstadt.

Advancing and receding contact angles of deionized (DI) water obtained from a Barnstead Micropure clean water system of Thermo Scientific were measured on the films. First, an initial droplet of 5 µL was placed on the surface. The needle was then immersed in the droplet, centered, and 12 µL of water was dispensed with a flow rate of 0.1 µL/s. After reaching the maximum volume (17 µL), the dosing stopped, the needle was centered again, and the water was withdrawn by the same flow rate of 0.1 µL/s. Throughout the complete procedure, a video of the drop was taken with a frame rate of approx. 3 fps. Advancing and receding contact angles and drop base diameters were determined from the video using the DataPhysics software SCA202 V. 4.1.13. On all prepared films, two drops were measured at least.

#### 2.2.3 Nanoparticles Formation and Characterization

##### 2.2.3.1 NPs Preparation

**FIGURE 2:**
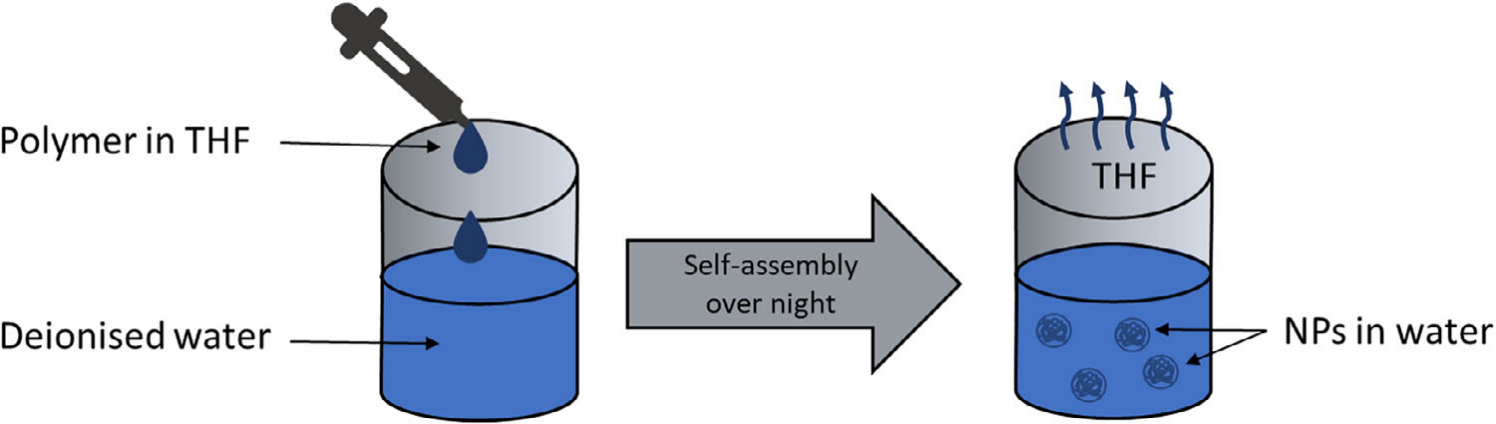
Scheme for the nanoprecipitation of PMTC to form NPs. First, the polymer dissolved in THF was slowly added to DI water and stirred overnight. At this time, the organic solvent evaporates and NPs are formed.

NPs were prepared by nanoprecipitation. Factors such as water‐solvent miscibility and polymer nature can affect the progress of the nanoprecipitation technique, Figure 2 [48]. Therefore, a suitable solvent must be carefully selected. Since THF was used as one of the common solvents, used for the analysis of PMTC, it was chosen for this process. Briefly, 5 mg of PMTC was dissolved in 1 mL of tetrahydrofuran (THF) and added dropwise into 10 mL of DI water under constant stirring at 500 rpm. The uncapped solutions were left stirring overnight to allow for complete evaporation of THF. The final NPs concentration was 0.5 mg/mL. After filtration through 0.22 µm cellulose filters, samples were analyzed for size and zeta potential (Table S3 and Figure S4).

##### 2.2.3.2 Dynamic Light Scattering (DLS) and Zeta Potential Measurements

Dynamic light scattering was used to determine NPs size using a Zetasizer Nano spectrometer (Malvern Instruments Ltd.) equipped with a 633 nm laser at a fixed angle of 173° and a Wyatt DynaPro DLS Plate Reader (Wyatt Technology Corporation, USA). Samples were equilibrated for 30 s at 25°C before measurement. A Zetasizer Nano spectrometer was also used to measure the zeta potential of the NPs. All samples were measured in triplicate. NPs were analyzed at a concentration of 0.5 mg/mL.

##### 2.2.3.3 NPs Stability in Fetal Bovine Serum (FBS)

Stock solutions of polymeric nanoparticles were prepared at 1.0 mg/mL in DI water. A stock solution of 20% FBS was prepared by diluting the original solution 1:5 with DI water. The polymer solutions (3 mL) were mixed with 3 mL FBS to reach a 10% solution, and samples were measured in the Zetasizer at *t* = 0, 1, and 24 h to assess the stability of NPs.

##### 2.2.3.4 NPs Stability at Different pH

Stock solutions of polymeric nanoparticles were prepared at 0.5 mg/mL in DI water. Stock solutions of citrate buffer (pH = 4), sodium phosphate buffer (pH = 12), and phosphate‐buffered saline (PBS) (pH = 7.4) were prepared. The polymer solutions (100 µL) were mixed with these buffered solutions, and samples were measured in the Zetasizer at t = 0, 30 min, 3 h, and 24 h to assess the stability of NPs.

##### 2.2.3.5 Transmission Electron Microscopy (TEM) Measurements

Prior to use, all TEM copper grids (carbon film 200 mesh copper (EM Resolutions)) were glow‐discharged at 15 mA for 20 s. NP suspensions (10 µL, 0.5 mg/mL) were added to the grid and left for 5 min, after which time the excess sample was removed. Subsequently, an aqueous solution of uranyl acetate (10 µL, 2 wt.%) was applied to the grid as a negative stain and left for 30 s, after which time the excess staining agent was removed. Analysis was performed using a FEI Biotwin‐12 TEM fitted with a digital camera.

#### 2.2.4 Coumarin Encapsulation

Coumarin‐6 (Cou6) Solution (0.5 mg/mL) was prepared in THF. Polymer (5 mg) was weighed into a vial and dissolved in the Cou6 solution (1 mL). These polymer solutions were subsequently added dropwise into DI water (10 mL) whilst stirring at 500 rpm. Vials were left under stirring overnight to enable solvent evaporation, and the produced NPs‐dye dispersions were filtered through a 0.22 µm filter. Cou6 was dissolved in DI water, without any polymer, in order to prepare a blank, and filtered through a 0.22 µm filter. particle sizes and Z‐potential were determined using DLS. In addition, the apparent water solubility of the dye was qualitatively determined using fluorescence spectrophotometry, by measuring the fluorescence intensity of the NPs‐dye dispersions at excitation wavelength λ = 460 nm and emission wavelength λ = 500 nm:

(1) $$\Delta F\%=\frac{\Delta F}{F}=\frac{\left(F_{\mathrm{NPs}}-F_{\mathrm{Cou}6}\right)}{F_{\mathrm{Cou}6}}\times 100$$

*F*_NPs_ = fluorescence signal of NPs formulation with encapsulated Cou6, normalized by the polymer F. *F*_Cou6_ = fluorescence signal of the free dye in water

#### 2.2.5 Enzymatic Degradation/Hydrolytic Enzymatic Assay

In order to evaluate the enzymatic degradability of the NPs, DLS was used as a quick and reliable technique to elucidate the behavior of the prepared NPs in the presence of a common lipase. By keeping the value of the attenuator stable, the change in the derived counter rate after enzyme addition was monitored. The derived count rate is a calculated parameter in the Zetasizer Nano software that is representative of the scattering intensity. The derived count rate was calculated from the measured count rate divided by the attenuation factor, meaning that when NPs losses occur due to sedimentation, the count rate will subsequently decrease [49].

For the degradation measurement of the NPs without an encapsulated dye, Lipase from porcine pancreas, Type II (≥125 units/mg protein (using olive oil (30 min incubation)), 30–90 units per mg protein (using triacetin)) was used. A solution of the enzyme at 10 mg/mL in PBS was prepared. 50 µL of this solution was added to 250 µL of NPs (at a concentration of 0.5 mg/mL in water, as mentioned previously). The enzymatic degradation was observed within 48 h at 25°C by DLS and zeta‐potential measurements.

For the degradation of the NPs with an encapsulated dye, Nile‐Red was coprecipitated with PMTC as discussed for the encapsulation study of Cou6. An Infinite M Plex, multimode microplate reader, monochromator optics were used as a spectrometer to allow for the determination of the fluorescence of a high number of samples. Three aliquots of each NP‐solution were individually mixed with a 0.02 m saline solution as a reference, or with an enzyme solution of lipase with a concentration of 10 U/mL in 0.02 m saline as a degradation sample. For each solution (3 reference, 3 enzyme), three aliquots of the three reference and degradation samples were placed in the microplate reader to allow for a high statistical certainty (following the method of triplets of triplets). The plate reader was heated to 37°C, and the measurement started after 5 min of equilibration time. Choosing an excitation wavelength of 550 nm, an emission wavelength of 630 nm, a gain of 100, and a number of flashes of 15, the fluorescence of each data point was used as settings for the measurement.

#### 2.2.6 Cytotoxicity Assays

##### 2.2.6.1 In Vitro Cell Culture

The human intestinal epithelial adenocarcinoma cell line Caco‐2 was obtained from the American Type Culture Collection (ATCC) and used between passages 40 and 50. Cells were cultured in Dulbecco's Modified Eagle Medium (DMEM) supplemented with 10% Fetal Bovine Serum (FBS) at 37°C in a humidified incubator with 5% CO_2_. Cells were routinely grown in 75 cm^2^ culture flasks to 70% confluence.

##### 2.2.6.2 In Vitro PrestoBlue Metabolic Activity Assay

Cellular metabolic activity was measured using the PrestoBlue viability assay (Thermo Fisher Scientific) as an indication of cytotoxicity. Caco‐2 cells were seeded at 1 × 10^4^ cells per well in 96‐well plates and cultured for 24 h. Cells were exposed to treatments (0.5 mg mL^−1^ NP concentration for all cell experiments unless stated otherwise) in 100 µL phenol red‐free DMEM for 24 h. Triton X‐100 was applied at 1% (v/v) as a positive cell death control, and medium alone was used as a negative control. Following the exposure period, treatments were removed and cells incubated with 100 µL 10% (v/v) PrestoBlue reagent per well, diluted in phenol red‐free medium for 60 min. The resulting fluorescence was measured on a Tecan Spark 10 m plate reader at an excitation wavelength λ = 560 nm and emission wavelength λ = 600 nm. Relative metabolic activity is calculated from PrestoBlue data by setting values from the negative control as 100% and positive control values as 0% metabolic activity according to the following equation:

(2) $$\begin{array}{ccc} & & Relative\;metabolic\;activity\\ & & \;=\left(\frac{\left(x-Positive\;control\right)}{\left(Negative\;control-Positive\;control\right)}\right)\times 100\%\; \end{array}$$

##### 2.2.6.3 In Vitro LDH Release Test

To study plasma membrane damage in vitro, the extracellular release of lactose dehydrogenase (LDH) enzyme was assessed using the LDH release assay (Sigma–Aldrich, TOX7 kit). As above, Caco‐2 cells were seeded at 1 × 10^4^ cells per well in 96‐well plates and cultured for 24 h. Cells were exposed to treatments in 100 µL phenol red‐free DMEM and received either polymeric formulations, 1% Triton X‐100 to induce cell lysis, or DMEM only to serve as the vehicle control. Following 24 h exposure, 50 µL of supernatant per well was sampled and transferred to a new 96‐well plate for the detection of LDH released extracellularly. LDH detection solution was prepared according to the manufacturer's instructions, and 100 µL detection reagent was added per well to the 50 µL of supernatant sample. The solution was incubated at room temperature, protected from light for 25 min, and the absorbance of the resulting solution was measured at 490 nm on a Tecan Spark 10 m plate reader. Relative LDH release was calculated by setting the absorbance signal of 1% Triton X‐100, assumed to generate full cell lysis, as 100% LDH release and the background signal generated by the LDH detection solution alone as 0%.

##### 2.2.6.4 Cellular Internalization Experiments

Cellular uptake of NPs on Caco‐2 cells was investigated using live cell fluorescent microscopy. Caco‐2 cells were seeded at 4∙10^4^ cells per well in 24‐well plates and cultured for 24 h. After 24 h, they were exposed to the 100 µL Cou6 encapsulated NP‐9‐8 NP solutions diluted in phenol red‐free DMEM for 24 h, followed by three washes with PBS to remove particles. Medium alone was used as a control. Cell nuclei were then stained with 10 µg mL^−1^ Hoechst 33342 applied in PBS for 30 min. The staining solution was then removed, and cells were washed twice with PBS. Cells were imaged on an inverted Thermo Fischer EVOS M5000 Imaging System on 4′,6‐Diamidin‐2‐phenylindol (DAPI) and cellulose filters 0.22 µm. Images were processed using ImageJ software (Version 7.1).

## 3 Results and Discussion

### 3.1 Polymer Synthesis and Characterization

In this work, different PMTC samples prepared by RROP were formulated into NPs. For the sake of simplicity, the samples are named by their molar mass and density of branches (DB). For instance, PMTC‐7‐9 has a molar mass of 7 kg/mol and a DB of 9%. The synthesis and details of all polymers are described in the Supporting Information (Supporting Information, including Table S1). Following the necessary resynthesis of polymer batches, some polymers, like PMTC‐28‐17 and PMTC‐27‐17, were considered equal and used interchangeably in the study. Please note that branching and molar mass in RROP solely correlate with the conversion without synthetic levers to promote or suppress the branching reaction. All polymers hence contain a mixture of short‐chain branching from intramolecular H‐transfer and long‐chain branching from intermolecular H‐transfer [46]. For that reason, low molar mass samples with a low DB and high molar mass samples with a high DB were readily accessible and hence chosen for this study.

**FIGURE 3:**
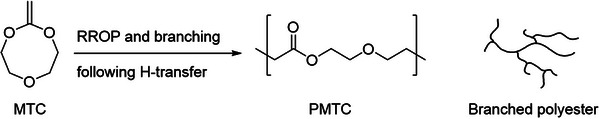
Synthesis scheme of the Radical ring‐opening polymerization of 2‐Methylene‐1,3,6‐trioxocane (MTC) toward PMTC. The ether function increases chain flexibility and the overall hydrophilicity of the polymer [50]. Following a radical polymerization mechanism, the RROP yields a branched polymer exhibiting a combination of short‐ and long‐chain branching [46, 51].

In various reports, MTC was reported to be the most hydrophilic monomer, rendering PMTC the most hydrophilic, or more precisely, the least hydrophobic PCKA (Figure 3) [5, 9, 11]. However, literature suggests that molar mass and the DB impact the polymer properties of other PCKAs and thus potentially also the hydrophilicity [10, 51, 52]. To quantify this hydrophilicity, contact angle measurements and solubility tests were performed.

**FIGURE 4:**
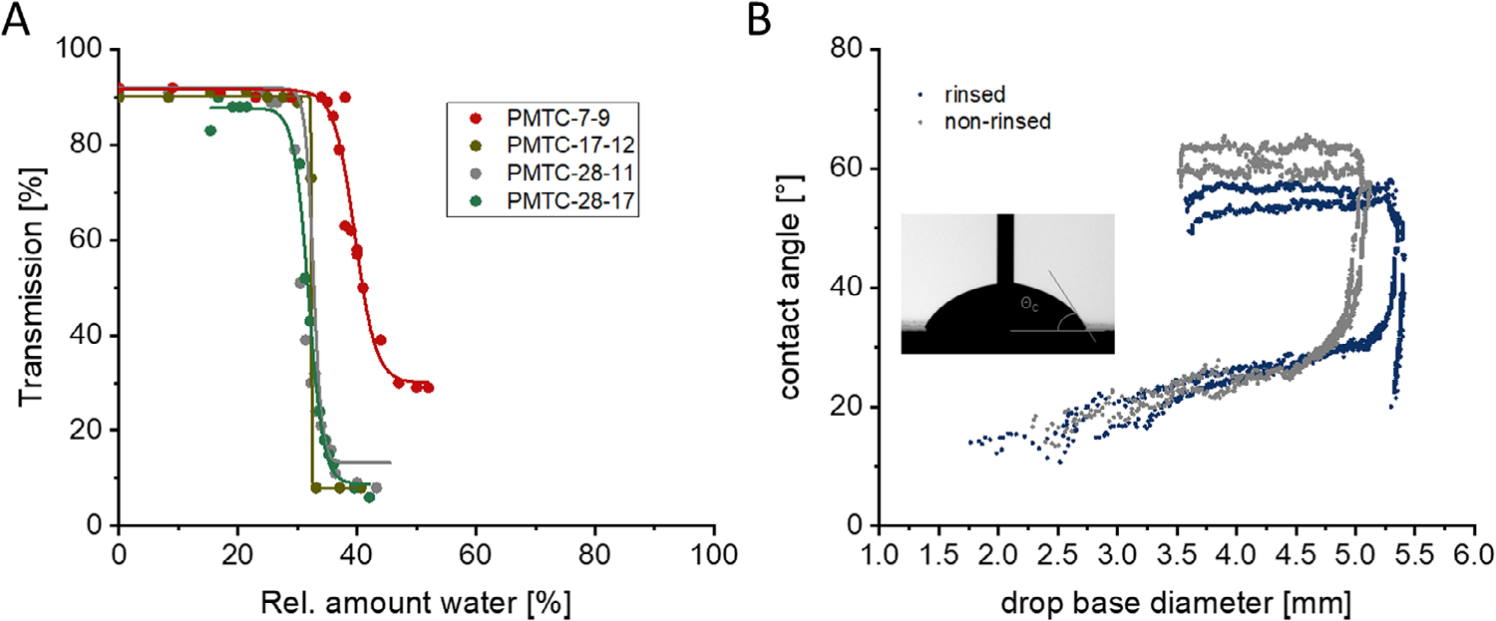
(A) UV–vis Transmittance of PMTC samples with varying molar mass and DB in various THF/water mixtures. All samples precipitate between 30% and 40% relative amount of water in the solvent mixture. The sample with the lowest molar mass and DB appears to precipitate at a slightly higher water content, but it also formed a less turbid solution. (B) Contact angle measurements of a thin film of PMTC‐7‐9, which was prepared by spin‐coating. One sample was rinsed with water (blue), the other one was measured without rinsing (gray). The graph shows the incoming and retracting traces.

In the first step, the hydrophilicity of PMTC was quantified by solubility tests in THF‐water mixtures. Polymers with varying molar mass and DB were first dissolved in THF before the polarity of the solvent mixture was increased stepwise by adding water. When the polarity, i.e., water content, exceeded a critical level, the polymer precipitated and the solution turned turbid. By measuring a decreasing transmittance, the precipitation point of the polymer was determined. It was shown that a comparable point of turbidity, independent of molar mass and DB, was observed for all samples (Figure 4A). In the range of 30–40 vol% water, the solution turned turbid following the precipitation of polymer from the previously transparent solution. Whilst the samples with the highest molar mass and DB, namely PMTC 17‐12, PMTC 28‐11, and PMTC 28‐17, precipitated with a sharp decrease in transmittance at only 30 vol% water, PMTC‐7‐9, with a lower molar mass and dispersity, differed slightly. This sample started to precipitate at approximately 40 vol% water, and the turbidity of the solution also did not decrease to the same extent. Following the Flory‐Huggins theory, a number of PCKAs (incl. PMTC), similar to the majority of polymers, were reported to be more soluble in apparent non‐solvents at lower molar mass. In light of this, the effect of precipitation of PMTC‐7‐9 at a higher water content could be explained [51, 53]. Other than that, no significant impact of branching or molar mass on the solubility was observed, indicating that neither characteristic are not significantly impacts the hydrophilicity of PMTC. Precipitating between 30 and 40 vol% of water also indicates a relatively low overall hydrophilicity of PMTC.

In order to further quantify the hydrophilicity of PMTC, contact angle measurements were performed as well. A polymer film of PMTC‐7‐9 was used for these measurements, as it was found to be the most hydrophilic PMTC sample. After test measurements of the contact angle on the spin‐coated polymer, water stains were found on the surface. To remove this artefact and allow for saturation of the surface with water, an additional washing step was implemented. Since the contact angle measurements are not only impacted by the polymer's hydrophilicity, but also the surface smoothness of the polymer film, this washing step also helped to further validate the measurement. However, a surface characterization beyond the progressing contact angle was beyond the focus of this study.

The advancing contact angle for PMTC was in the range of 50°–65° before and after rinsing the surface (see Figure 4B). Whilst the observed contact angle for the rinsed sample was slightly lower than the contact angle of the non‐rinsed sample, the deviation of the advancing contact angle was less than 15°. While the measured contact angle fluctuated between 54° and 64° for all measurements, an average of 58 ± 4° was determined (Figure S3 and Table S2). Considering the deviation before and after the rinsing and statistical errors, it can be assumed that the value of the contact angle of PMTC is in the range of 60 ± 10°. Considering that the polymer is not water‐soluble, it is surprising that the observed contact angle is in the range of hydrophilic surfaces. Comparing this value to the literature, this contact angle is lower than poly(ε‐caprolactone) (PCL), with a contact angle of roughly 100°, and poly(ethylene terephthalate) (PET) or poly(dimethyl siloxane) (PDMS) with contact angles of 70° and 90°, respectively [42]. According to literature, for a PCL‐variant with a similar structure as PMTC, the additional ether function also increased the hydrophilicity of the polyester, but the polymer remained insoluble in water [50]. Polydioxanone, as a polymer that is structurally closest to PMTC, was reported to have a comparable contact angle to PMTC with 52° [54].

The obtained contact angle is thus comparable to conventional, commonly used polymers. Moreover, a large hysteresis between receding and advancing contact angle of roughly 50° hints toward an interaction between polymer and water, which is necessary for the self‐assembly in nanoprecipitation [42]. In our case, this means that a nanoprecipitate of the hydrophobic PMTC was likely to be stabilized by hydrophilic groups on the surface, enabling nanoprecipitation as a suitable formulation method.

### 3.2 NP Formation by Nanoprecipitation

With suitable polymers in hand, nanoprecipitation was performed. For that, PMTC‐27‐17, PMTC‐9‐8, and PMTC‐10‐4 were selected for further studies as they cover a polymer with a high molecular weight and high DB, and two polymers with low molecular weight and different DB were tested for their ability to form NPs from nanoprecipitation.

Following a published protocol, all synthesized polymers proved their self‐assembly capabilities, regardless of the molar mass and the DB [38]. All polymers formed NPs with sizes in the range of 100‐190 nm, and PDI values of 0.25 and 0.10 indicated an acceptable range of homogeneous NPs (see Table 1) [55, 56]. However, a trend to NPs with larger diameters at a higher DB can be suggested as the size increases from around 100 nm for 17% DB over to 130 nm for 8% DB to 190 nm for 4% DB. This can be reasoned as the branching groups shorten the overall length of the polymer chain, and a lower DB means comparatively long polymer chains. Thus, more polymer material is required to stabilize the particles, and the size increases [57, 58]. However, since the polymer conformation and density of the particles were not looked into in detail, this remained a speculation. A clear trend for molar mass could not be seen or may have been overshadowed by the branching effect. On the other hand, the differences in molar mass do not allow for a definite statement on the branching, which should be seen as indications instead. To gain statistical certainty about the discussed effects, a greater number of polymers have to be analyzed, but this was not the focus of this work.

**TABLE 1:** Compilation of DLS data obtained from different PMTC‐NPs prepared by nanoprecipitation.

Polymer	Z‐Average [nm]	Peak diameter [nm]	PDI
PMTC 27‐17	100 ± 10	110 ± 10	0.11 ± 0.01
PMTC 9‐8	130 ± 10	150 ± 10	0.10 ± 0.06
PMTC‐10‐4	190 ± 10	190 ± 10	0.24 ± 0.33

*For all samples, the attenuator was 10.

**FIGURE 5:**
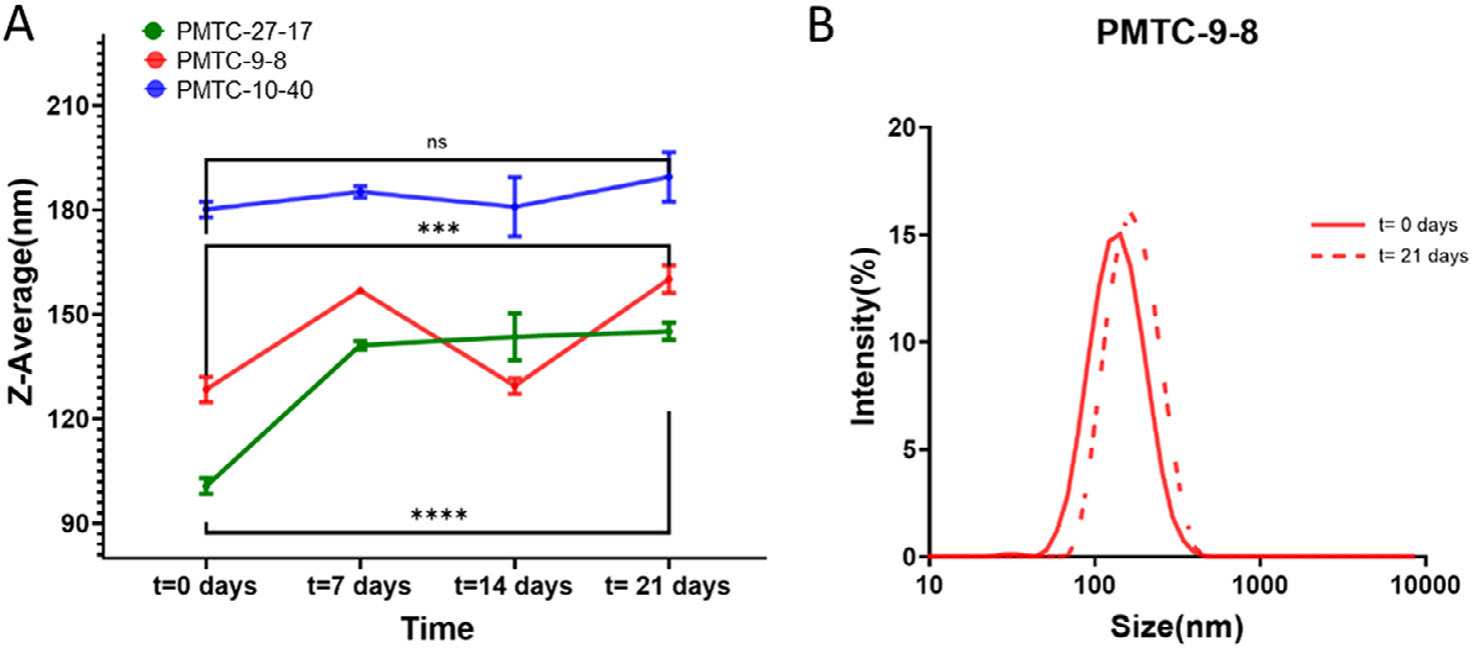
(A) Stability test and Z‐average of PMTC‐NPs from selected polymers at 0, 7, 14, and 21 days measured by DLS. (B) DLS traces for PMTC‐9‐8, at t = 0 and t = 21 days, where the z‐averages of these traces were used for part A of this figure.

To test the stability of the polymer samples, DLS measurements after 0, 7, 14, and 21 days were performed. As discussed above, a difference in the size of the NPs was observed, showing a possible link to the DB of the polymers. For PMTC‐10‐4, a comparable size throughout the experiment was observed. Starting with a size of roughly 180 nm, the size after 21 days was the same as at t_0_. For PMTC‐9‐8, an increase and a decrease in the size were observed, not following a distinct trend. Since the deviation within the experiments is within the range of 40 nm, this can be seen as an experimental error, and the NPs are considered long‐term stable throughout the storage time. Both other polymers, PMTC‐27‐17 and PMTC‐9‐8, showed a slight increase in size over time, from t_0_ to 7 days (Figure 5A, e.g.100–140 nm for PMTC‐27‐17, see also Table S4 and Figure S5). The NPs are probably swollen or have been reaggregated to form bigger NPs, but since the size did not increase from 7 days to 21 days, these NPs can be considered stable. Looking at the underlying DLS trace, the increases in sizes were marginal (Figure 5B), all had a low PDI below 0.25 (see Table S3), proving a shelf‐lifetime of 21 days or more. It was less surprising that the NPs from polymers with shorter linear segments, i.e., higher DB, showed some rearrangement over time, as these were less heavily entangled than NPs from polymers with a low DB (PMTC 10‐4). An indicator of this rearrangement following chain mobility was found in PMTC‐9‐8, after 14 days, where a minor agglomeration was found in the DLS trace, which disappeared after 21 days (see Figure S6 and Figure 5B). Other than that, agglomeration could never be found, reiterating the stability of PMTC‐NPs from nanoprecipitation. The combination of PMTC being a hydrophobic polymer and the interaction between polymer and water observed in the contact angle measurements appears to stabilize the NPs.

In order to investigate the NPs by an additional method, transmission electron microscopy (TEM) was performed on all three polymers. Uniform, but much smaller NPs were observed (TEM gave 20–25 nm instead of 120 nm in DLS) directly after NP formation and 42 days later (Figure S14). This size difference could be due to swelling, but it is unlikely to be the sole reason, given the hydrophobicity of PMTC. Following the size difference of a factor of 6, the larger NPs in water likely disassembled into smaller NPs after drying on the TEM grid. As shown above, PMTC only has a hydrophilic surface, but is actually hydrophobic. Upon the transfer from water to the more hydrophobic air, the surface of the NPs requires less stabilization by a hydrophilic surface, and they consequently disassemble (see Figures S14 and S15).

An attempt to explain the behavior of PMTC‐NPs was made in Figure S15. However, with missing references from the literature of polymeric nanocarriers from PCKAs only and the reported challenges of the low T_g_ of macroscopic PCKA‐based particles in an earlier work, detailed studies should be considered in the future [59]. However, to the best of our knowledge, RROP‐based‐NPs with a CKA‐content above 20% were never formulated before, and the regular size of both samples: TEM and DLS, should be considered a first proof of concept of the use of PMTC‐NPs.

### 3.3 NPs Stability in Different pH Values

Stability in DI water is an important characteristic to determine the shelf‐time of nanoparticles and handy for research purposes, but does not reflect any natural or physiology‐relevant environment. Any biological environment will contain a complex mixture of proteins, sugars, and other molecules. Stability in 10% fetal bovine serum (FBS) was checked on NPs from all polymers for 24 h. All showed some increase in the PDI, and the correlation function showed an onset of precipitation, indicating possible protein‐polymer interactions (Table S5 and Figure S6). Short term‐stability tests at different pH values were then performed to assess the behavior of the NPs in different pH environments [60, 61]. In order to mimic tumor environments, an acidic citrate buffer with a pH of 4 was used [62, 63, 64]. For neutral pH‐values, a phosphate‐buffered saline (PBS) buffer at pH 7.4 was used to mimic blood, cell culture media, and human fluids [65, 66, 67]. A sodium phosphate buffer representing an alkaline pH value of 12 was used to mimic the environment of bacterial interference [68, 69].

**FIGURE 6:**
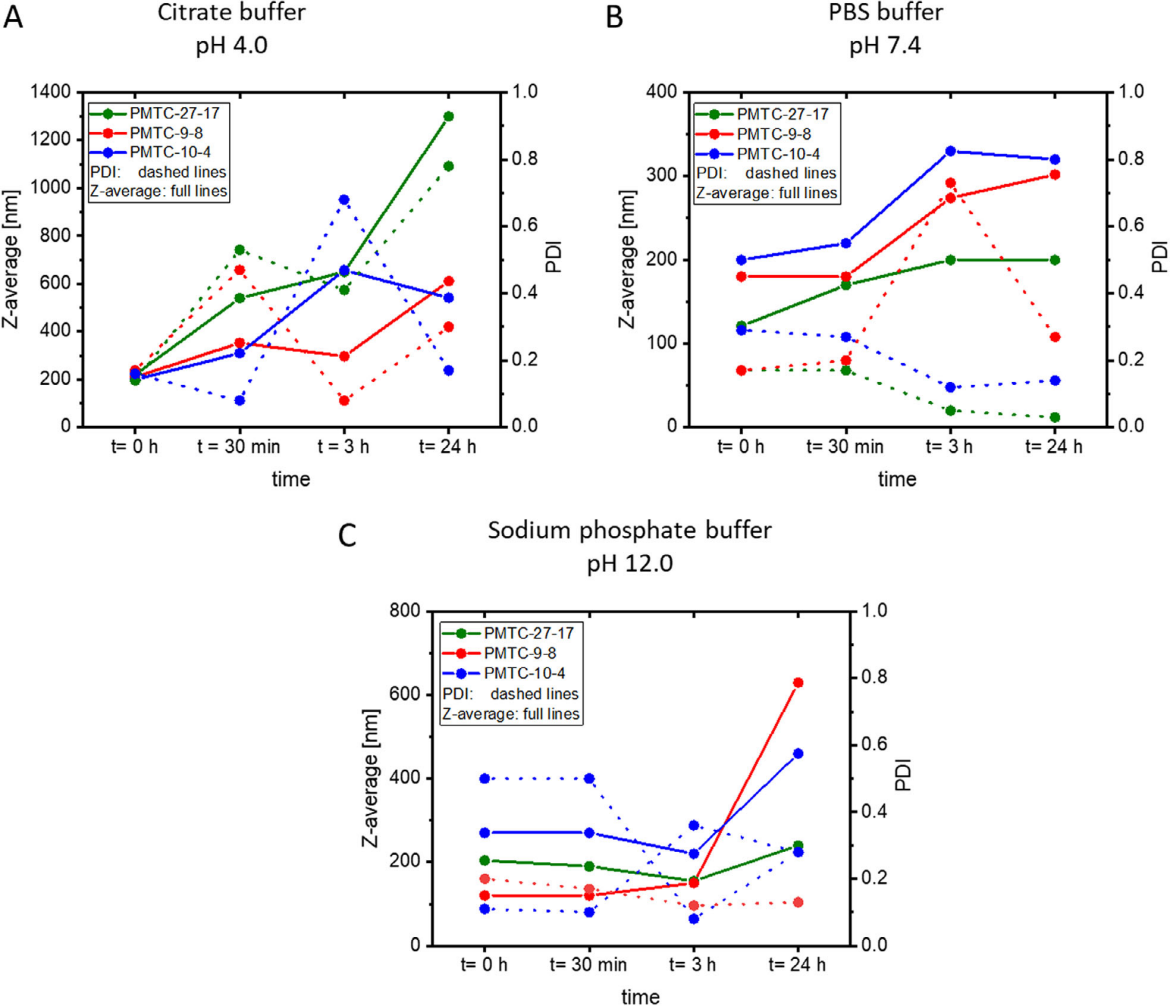
Sizes from DLS‐data (z‐average) at different time points for PMTC‐27‐17, PMTC‐9‐8, and PMTC‐10‐4 at different pH values (A: 4.0, B: 7.4, C: 12.0). The full line represents the Z‐average obtained from DLS and the dashed line the PDI from these measurements. Note that a different axis for the size of the NPs was also used, showcasing the stability at different pH values. The used color code is the same as for previous figures.

In an acidic environment at pH 4.0 (Citrate buffer, Figure 6A; Table S6 and Figure S7), all NPs showed an increase in size for the first 3 h. After 24 h, the size of PMTC‐27‐17 increased from 200 nm to roughly 1300 nm, indicating NP disassembly, most likely due to hydrolysis of the PMTC. The hydrolysis would lead to the formation of hydroxy‐ and carboxylic acid groups, increasing the hydrophilicity of the PMTC‐homopolymer. This would lead to an enhanced swelling of the particles and ultimately to disassembly. The combination of unswollen, swollen, and disassembled NPs can be seen in the stark increase of the PDI from 0.14 to 0.78, demonstrating the disassembly of NPs following ester hydrolysis. For PMTC‐9‐8, the size increased from 200 to 600 nm, and the PDI increased from 0.17 to 0.30, also suggesting a steady disassembly until 3 h. For PMTC‐10‐4, a similar increase from 200 to 600 nm was observed; however, only a minimal change in PDI from 0.16 to 0.17 was observed. The observed PDI of 0.7 suggests previous disassembly. With that, the size difference between these data suggested a strong disassembly process at pH 4, making them viable NPs for drug delivery in tumor or stomach tissues.

NPs at pH 7.4 (PBS Buffer, Figure 6B; Table S7 and Figure S8) followed the discussed trend from DI water and showed an initial swelling phase followed by a steady size. NPs from PMTC‐27‐17 increased their size from 120 to 200 nm at a decreased PDI of 0.17 and 0.03. The size of PMTC‐9‐8 changed from 180 to 300 nm with a PDI change from 0.17 to 0.27, and the size of NPs from PMTC‐10‐4 changed from 200 to 320 nm with a decrease in PDI from 0.29 to 0.14. Despite NP swelling in the range of 80–120 nm, the PDI of the NPs decreased in some samples, suggesting some reorganization and swelling of the nanoparticles, but no significant disassembly. It is unclear what drives the intermediate spike in PDI observed at 3 h in pH 7.4 conditions for PMTC‐9‐8; however, the final measurement at 24 h demonstrates this value returning to 0.3, and thus an artefact in the measurements may be responsible.

At an alkaline pH of 12 (sodium phosphate buffer, Figure 6C; Table S8 and Figure S9), an initial stable phase of comparable NP‐size was observed for all NPs, which was only steady for PMTC‐27‐17 from 200 to 220 nm at a PDI of 0.20 and 0.13, increased from 120 to 600 nm at an increased PDI of 0.11 and 0.28 for PMTC‐9‐8 and from 280 to 450 nm for PMTC‐10‐4 at a PDI of 0.50 to 0.28. Comparing the correlation functions, all samples showed a disassembly resulting from the hydrolysis of PMTC observed in the correlation function (Figure S9). Considering these results, a bacterial interface might be a suitable environment for drug release using PMTC‐NPs. At pH 12, a similar NP disassembly behavior is observed relative to an acidic pH value. Interestingly, comparatively low PDI values were observed, suggesting a homogeneous disassembly.

### 3.4 Model Drug Encapsulation Screening

To investigate the use of these polymeric NPs as drug delivery carriers, an initial encapsulation study was conducted using a hydrophobic compound (logP = 4.9), Coumarin‐6 (Cou6), as a model drug, following previous protocols by exploiting its fluorescence properties [42, 70, 71]. Since Cou6 is hydrophobic, it is an ideal system to mimic the behavior of lipophilic active pharmaceutical ingredients such as antibacterial or antioxidant active drugs [72]. The relative amount of encapsulated Cou6 in PMTC‐NPs is shown in Figure 9.

**FIGURE 7:**
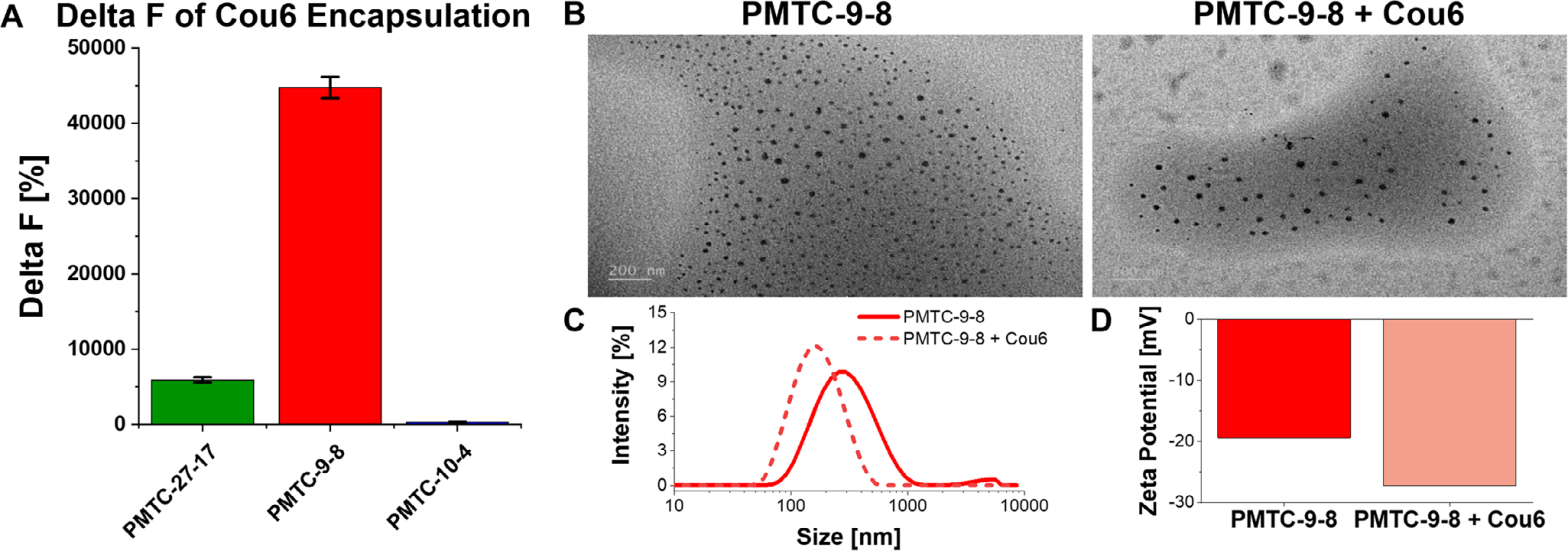
(A) ΔF% ranking of Cou6‐loaded NPs of the studied PMTC polymers in relation to empty NPs. (B) TEM images of PMTC‐9‐8 alone and loaded with Cou6, scale bars = 200 nm. (C) DLS traces showing the size distribution of PMTC‐9‐8 with and without Cou6 encapsulated. (D) Graph illustrating the zeta‐potential values of polymer NP‐9‐8 and polymer NP‐9‐8 encapsulated with Cou6.

Cou6 was co‐nanoprecipitated with and without polymers (Figure 7A; Table S10 and Figures S12 and S13). The change in fluorescence ΔF% was used as a fast and semi‐quantitative technique to determine the apparent solubility of the loaded Cou6 in water. The relative increase in fluorescence signal (hydrophobic environment = encapsulated), reported as ΔF% values, could be directly related to the encapsulation of Cou6. The best performing NP in terms of encapsulation was PMTC‐9‐8, which demonstrates a ΔF% of approx. 45 000% (Figure 7A). PMTC‐27‐17 NPs demonstrate some ability to encapsulate Cou6 with a ΔF% of roughly 700%; however, low‐to‐no Cou6 encapsulation was exhibited with PMTC‐10‐4 NPs (ΔF% < 100%). These results suggest that lower molar mass combined with medium DB levels is optimal for efficient encapsulation. A similar trend was reported in an earlier study for the comparison of NPs from PEG‐PCL and PEG‐PMDO, where branching appeared to enhance the loading of Curcumin due to the additional side‐groups from branching [73]. However, as no clear correlations could be identified, no structural arguments could be seen as apparent, and more detailed studies must be conducted to assign clear correlations and causations to the DB and molar mass.

Since the NPs of PMTC‐9‐8 demonstrated the highest amount of encapsulated Cou6 of the three polymers, the drug‐loaded NPs of this polymer were investigated further. DLS, Zeta Potential, and TEM studies were performed to investigate whether the size, shape, and surface charge of the Cou6‐loaded NPs differ from the non‐loaded NPs (TEM in Figure 7B). In line with previous investigations, the TEM showed the disassembly of the NPs into smaller NPs upon drying. After Cou6 encapsulation, DLS suggested that the sizes of the NPs remain within the same range, with a small notable shift to smaller sizes (Figure 7C), and the zeta potential values dropped from around −19 to −27 mV (Figure 7D). This change of surface charge might be due to some reassembly of the polymer chains within the NPs due to the interaction of polymers with the dye, but they are within the expected range and generally show that the NPs were not impacted by the dye loading [42]. This can now serve as a first lead when designing PMTC‐NPs for other drugs as well, even though the optimal loading may be different for each polymer‐drug combination.

### 3.5 Degradation Studies

The enzymatic degradation of PMTC‐copolymers has been reported previously in the literature, and recently, even the biodegradation of the PMTC‐homopolymer following the OECD‐F‐standard [5, 13, 21, 26, 27, 42]. After successfully covering the dye‐encapsulation of the PMTC samples, the enzymatic degradation of the NPs with and without an encapsulated dye was performed. To track the degradation of the crude samples, DLS measurements and zeta‐potential measurements were performed to track the change in the size and surface charge, respectively. To track a potential release of a drug upon enzymatic degradation, previously established fluorescence release studies with Nile‐red were conducted as well. The assay exploits that Nile Red shows an enhanced fluorescence in a more hydrophobic environment (= encapsulated state). Upon degradation of PMTC, Nile red gets released into the aqueous phase, losing its fluorescence, allowing for tracking the NP degradation by following fluorescence intensity [27].

**FIGURE 8:**
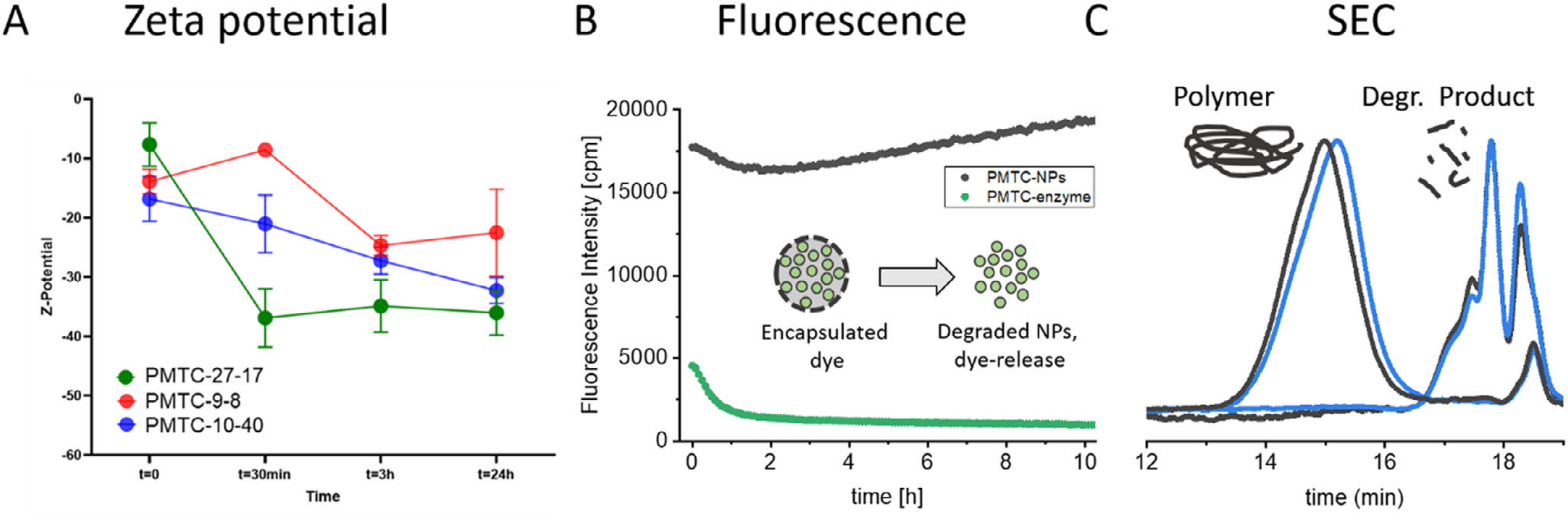
Results from the degradation experiments using lipase: (A) Zeta‐potential measurements at the respective time points, (B) Fluorescence‐intensity of the polymeric NPs with and without enzyme (cartoon: complete NP with encapsulated dye that becomes free dye), (C) SEC‐elugram from the polymer PMTC 27‐17 before (black trace) and after enzymatic degradation (blue trace) illustrating a complete degradation. The traces show two injections of the same sample (doublet measurements).

The z‐average decreased for all NPs independent of the molar mass and DB already after 3 h, indicating a negative surface charge caused by the degradation of the ester bond (Figure 8A; Figures S10 and S11). Thus, a first indication toward an enzymatic degradation of the polymeric NPs was observed and was validated by an increasing PDI and Z‐average as depicted in Section S3. Degradation of NPs without an encapsulated dye was proven. Degradation of dye‐loaded NPs was performed next. Very conveniently, the control of this study allowed us to validate the stability of the NPs in saline solution. As indicated in Figure 8B, the dye‐loaded polymeric nanoparticles showed no decrease in the fluorescence intensity over 10 h at 37°C in PBS, proving that no dye leached out of the NPs. When the lipase was added, the fluorescence intensity immediately decreased, indicating dye release due to the degradation of the PMTC. In order to validate the degradation of the PMTC‐chains with SEC, the same procedure was applied to nanoparticles without the Nile red to avoid interference with the RI‐detector of the SEC setup. As depicted in Figure 8C, the freshly prepared NPs were still intact and did not show any hydrolysis as shown by the uniform, monomodal SEC‐trace. When the NPs were treated with the lipase, the polymer degraded and hence the polymer peak disappeared, leaving only traces of oligomeric side products and residual proteins of low molar mass to be detected by the SEC. Degradability of the PMTC‐NPs was hence proven with and without cargo by DLS, Fluorescence, and SEC.

### 3.6 Toxicity Testing

After proofing the enzymatic degradation of PMTC, cytotoxicity studies were performed. PMTC‐bearing polymers proved to be non‐toxic on various occasions [21, 26, 27] which is why the toxicity studies of this work were also conducted as a preliminary experiment for the following cell‐uptake studies. A potential impact of branches and molar mass of the PMTC‐NPs was checked by cytotoxicity tests with Caco‐2 intestinal epithelial cells. Caco‐2 cells are often used in polymeric cytotoxicity assays to study the translocation of nanomedicines across the intestinal barrier, including studies of absorption and metabolism [42, 74, 75, 76]. A broader picture of the cytocompatibility was obtained by conducting both toxicity studies on both PMTC and its degradation product. The degradation product was obtained from accelerated degradation under alkaline conditions [11]. In order to test biocompatibility, two test assays were performed: (i) PrestoBlue assay to test for metabolic activity and (ii) LDH release assay to test for potential plasma membrane damage.

**FIGURE 9:**
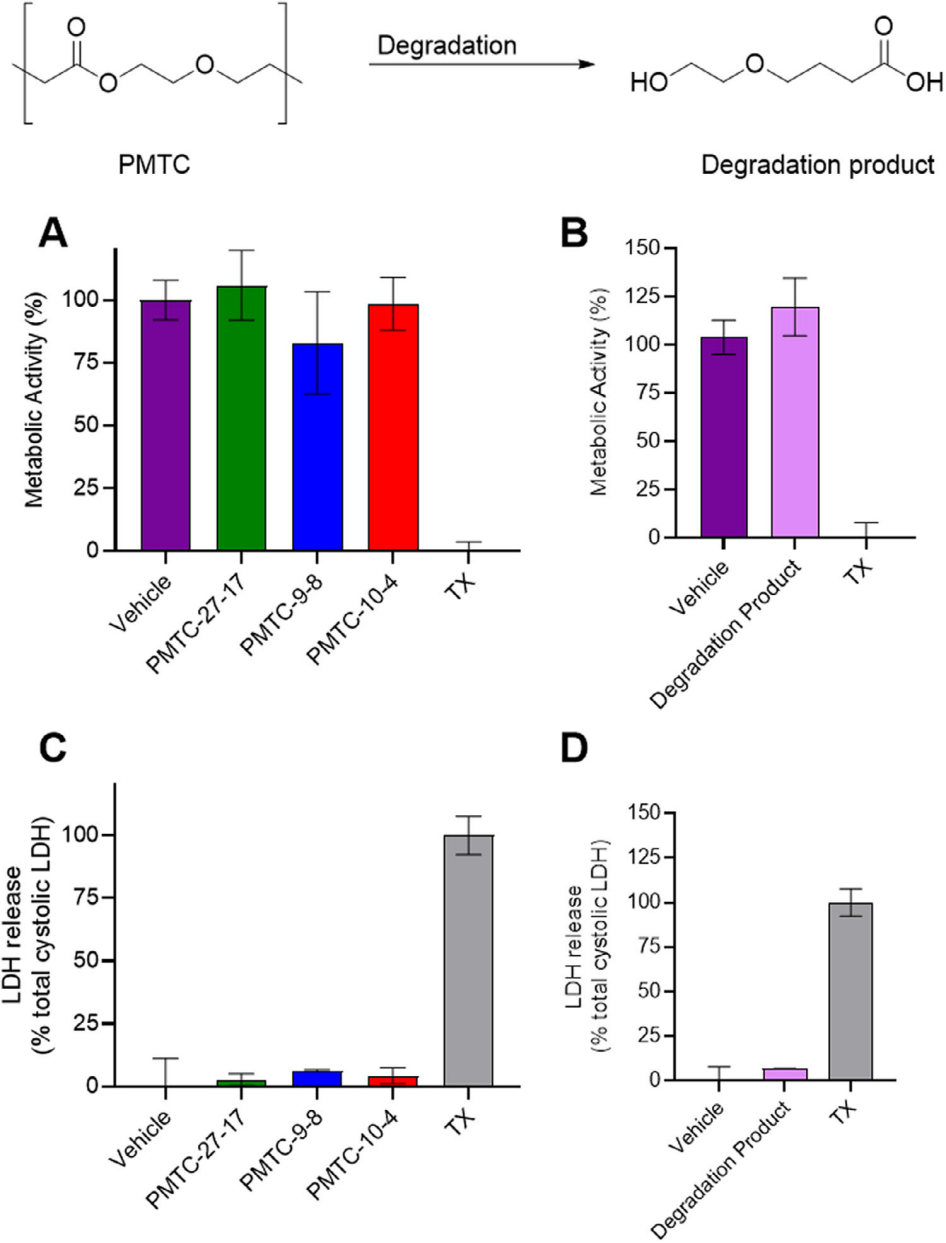
In vitro cytotoxicity of polymeric NPs (A, C) and PMTC degradation product (B, D) on Caco‐2 intestinal epithelial cells. Cytotoxicity was assessed via the following. For A, B: metabolic activity using PrestoBlue assay. For (C, D) LDH release assay. Cells were exposed for 24 h to 0.5 mg mL^−1^ of polymeric systems and DMEM culture media as a vehicle control and 1% (v/v) Triton X‐100 (TX) as a cell death‐inducing control. Data presented as mean ± S.D. Both assays show coherent results.

Investigating the different PMTC‐NPs (Figure 9A) and the degradation product of PMTC (Figure 9B), no substantial decreases in metabolic activity ≤ 80% were observed. In contrast to TX as a cell‐death‐inducing negative reference, no toxicity was observed, suggesting PMTC‐NPs to be suitable candidates for biomedical applications. No significant differences were noted between PMTC groups with different DB and molar mass in terms of cytotoxicity measurements. In order to validate these data, in the next step, a test toward plasma‐membrane damage was conducted. As shown in Figure 9C,D, neither the PMTC samples nor the PMTC‐degradation product caused membrane damage, reiterating the suitability for PMTC as nanocarriers. As NPs of PMTC homopolymers and their degradation products were non‐toxic, independent of DB or molecular weight, this suggested a high feasibility of this polymer for drug delivery applications. These findings are in line with previous biocompatibility studies of PCKA‐based copolymer systems [21].

### 3.7 Cellular Internalization Experiments

In order to test the cell‐uptake and release of the prepared nanocarriers, Cou6‐loaded NPs from PMTC‐9‐8 were tested for their drug delivery applications using cell uptake experiments in intestinal epithelial cells.

**FIGURE 10:**
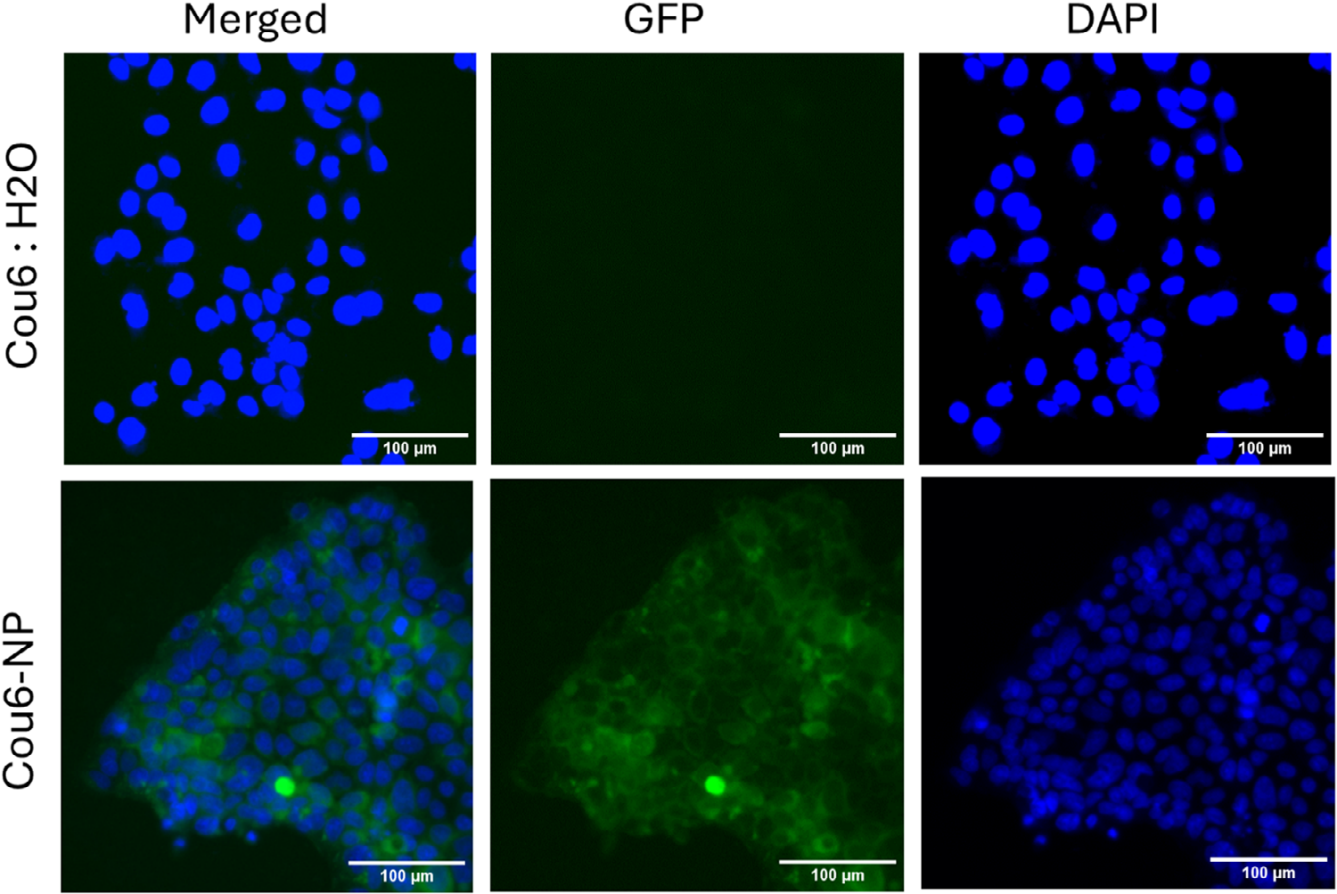
Cell imaging results demonstrating the cell uptake in Caco‐2 epithelial cells. Cells were incubated with the nanoparticle solution of polymer NP‐9‐8 encapsulating Cou6 at a concentration of 0.5 mg mL^−1^ (0.1 mg/mL for Cou6) for 24 h. A solution of Cou6 dissolved in water at a concentration of 0.05 mg/mL, which was used as a control. Images are representative of three independent repeats. A scale bar = 100 µm is being used.

Fluorescent microscopy was employed through the intracellular tracking of Cou6‐loaded PMTC‐9‐8 NPs. Following incubation with cells for 24 h, the intracellular presence of Cou6 fluorescent signal can be observed when delivered by PMTC‐9‐8 NPs (Figure 10A). This is contrasted by control experiments with Cou6 alone, which demonstrate the lack of Cou6 signal within cells (Figure 10B). Due to Cou6's negligible solubility in water, its fluorescence was only observed in the presence of a hydrophobic chemical environment, such as PMTC [77]. Together, these data indicate the successful delivery of Cou6 as a model drug to intestinal epithelial cells and enable future work with PMTC NPs as drug delivery vesicles to be conducted.

## 4 Conclusion

In this work, nanoparticles from P(cyclic ketene acetals) (PCKAs) homopolymers have been prepared and tested for their potential use in the biomedical field. The effects of branching, a known intrinsic side reaction of RROP, on the self‐assembly behavior are discussed for the first time. Whilst branching did not impact the hydrophilicity of PMTC, it altered the formulation properties. Whilst for all discussed polymers with varying DB and molar mass, nanoparticles could be prepared, increasing the DB leads to smaller NPs. All PMTC‐NPs generated are less than 200 nm in diameter and thus suitable from a size perspective for nanomedical applications. An optimum of Cou6 loading could be identified between molecular weight and DB has been found for PMTC NPs (PMTC‐9‐8). The presence of lower levels of branching (PMTC‐10‐4) was observed to generate the largest diameter NPs, which also possessed the longest‐term stability. This suggests that the longer linear sites enabled increased entanglements during nanoprecipitation that conferred higher colloidal stability. The data highlight the importance of controlling polymer structure and branching during RROP for generating subsequent polymeric NPs.

The PMTC homopolymer has been shown to form NPs using nanoprecipitation, which has the advantage of being a simple and cost‐effective procedure, and the generated NPs have shown stability for at least 42 days in neutral pH conditions. Moreover, the successful encapsulation of Cou6 as a model hydrophobic drug, combined with the non‐toxic nature of the NPs and evidence of NP uptake in vitro, highlights the promising ability of PMTC as a polymeric drug delivery system. Finally, in a wider setting, the results from this study aim to draw attention to the growing potential of RROP within chemical research and suggest the formulation of more PCKAs.

## Author Contributions

**Eleni Axioti**: methodology, validation, formal analysis, investigation, data curation, writing (original draft, review and editing) and visualization. **Fabian Mehner**: methodology, validation, formal analysis, investigation, data curation, writing (original draft, review and editing), visualization, funding acquisition. **Morgan Reynolds‐Green**: methodology, formal analysis, investigation, writing (review and editing). **Aniket Rahul Bukane**: formal analysis, investigation, data curation. **Robert Cavanagh**: conceptualization, methodology, visualization, writing (review and editing). **Günter K. Auernhammer**: methodology, formal analysis, investigation, writing (review and editing). **Stefan Michel**: methodology, formal analysis, investigation, writing (review and editing). **Vincenzo Taresco**: conceptualization, methodology, validation, writing (review and editing), visualization, supervision, project administration, funding acquisition. **Jens Gaitzsch**: conceptualization, methodology, validation, data curation, writing (review and editing), visualization, supervision, project administration, funding acquisition.

## Conflicts of Interest

The authors declare no conflicts of interest.

## Supporting information




**Supporting File**: mabi70094‐sup‐0001‐SuppMat.pdf.

## Data Availability

The data that support the findings of this study are available from the corresponding author upon reasonable request.
