# Supplementary material for: Nanoprecipitation and Drug Delivery with PMTC: Toward Biomedical Application of Polyesters from Radical Ring‐Opening Polymerization
Source: Macromol Biosci. 2025 Oct 19;26(1):e00432. doi: 10.1002/mabi.202500432 (PMC12829526; doi:10.1002/mabi.202500432)
Supplement: Supplementary file 1 — Supporting File: mabi70094‐sup‐0001‐SuppMat.pdf. [file MABI-26-e00432-s001.pdf]

Supporting information:

Nanoprecipitation and drug delivery with PMTC: Towards biomedical application of polyesters from radical ring-opening polymerisation

Eleni Axioti [a),#], Fabian Mehner [b,c), #], Morgan Reynolds-Green[a)], Aniket Rahul Bukane [b,c)], Robert Cavanagh [a)], Stefan Michel [b)], Günter K. Auernhammer [b)], Vincenzo Taresco* [a)] and Jens Gaitzsch* [b)]

[a] School of Chemistry, University of Nottingham, University Park, Nottingham, NG7 2RD, United Kingdom

[b] Leibniz-Institut für Polymerforschung Dresden e.V., Hohe Straße 6, 01069 Dresden, Germany

[c] Technische Universität Dresden, Faculty of Chemistry and Food Chemistry, Organic Chemistry of Polymers, 01069 Dresden, Germany

# E.A. and F.M. contributed equally

* Corresponding authors: gaitzsch@ipfdd.de (J.G.), Vincenzo.Taresco@nottingham.ac.uk

Keywords: RROP, Nanoparticles, drug delivery, polyester. Raw data DOI: 10.5281/zenodo.14051766

Table of Contents

1. Synthetic procedures and characterisation 2
1.1. 2-Chloromethyl-1,3,6-trioxocane (MTC-Cl) and 2-methylene-1,3,6-trioxocane (MTC) 2
1.2. Synthesis of PMTC 2
1.3. Hydrolysis of PMTC 3
1.4. Contact angle measurement 3
2. NPs formation and characterisation 5
2.1. NP filtration tests 5
2.2. Stability of NPs 6
2.3. Stability in different environments 7
3. Enzymatic degradation 10
4. Drug encapsulation 11
5. TEM-measurements 13
6. References 15

1. Synthetic procedures and characterisation

1.1. 2-Chloromethyl-1,3,6-trioxocane (MTC-Cl) and 2-methylene-1,3,6-trioxocane (MTC)

The synthesis of MTC and MTC-Cl was performed as reported in literature and is described in detail in section 1 of the SI.[1] Briefly, diethylene glycol, chloroacetaldehyde dimethylacetal and DOWEX WX2 were mixed, heated up to 120 °C and stirred for 3h. The product was distilled to obtain MTC-Cl as white crystallites. The chloroacetal was then dissolved in tert-butanol and potassium *tert*-butoxide was added, before the mixture was heated to 120 °C. The product was extracted with diethyl ether and distilled multiple times to gain MTC as a clear, transparent liquid.

1.2. Synthesis of PMTC

The synthesis of PMTC was followed by the thermal polymerisation reported in literature.[2] In brief, MTC and AIBN was mixed and degassed with a gentle argon stream for 15 minutes. The mixture was then stirred at 85 °C or 65 °C and quenched by cooling the mixture with ice and opening the reaction vessel. The product was worked up by precipitation in ice cold diethyl ether and solvent the product dried in a vacuum oven at 50 °C.

Table S 1:Compilation and assignment of polymers used in the manuscript with their respective reaction conditions, obtained conversion, molecular weight. The samples were named (PMTC-M_n-DB) according to their molecular weightand DB and for the assignment to the figure in the main text was used.

sample	m_{MTC} [g]	V_{tBuOH} [mL]	m_{AIBN} [mg]	t [min]	T [°C]	Conversion [%]	DB [%]	M_n [kg/mol]	Used for Figure*
PMTC-28-17	0.75	0.54	10.0	160	85 °C	89	16.6	28.1	2
PMTC-28-11	0.35	-	4.8	240	65 °C, MW	84	11.3	27.9	2
PMTC-17-12	0.35	0.90	4.8	1460	65 °C	82	12.0	16.7	2
PMTC-7-9	2.44	-	30.8	960	85 °C	50	9.3	7.4	2
PMTC-9-8	1.22	-	15.4	60	85 °C	73	7.5	9.0	4
PMTC-9-8-2	1.22	-	15.4	60	85 °C	47	8.0	9.0	4
PMTC-10-5	1.22	3.70	15.4	120	65 °C	27	4.7	10.0	4
PMTC-27-17	1.22	0.9	15.4	100	85 °C	86	16.8	27.0	4
PMTC-7-8	1.22	-	15.4	360	85 °C	47	8.0	7.0	8

*Experiment in the Figure: 2…solubility and contact angle measurement; 4…synthesis of nanoparticles; 8…biocompatibility of the hydrolysis product. Re-synthesis of PMTC-28-17 for FBS tests (section 2.3) resulted in PMTC-36-17, which is not listed separately.

1.3. Hydrolysis of PMTC

In order to test the biocompatibility of the degradation product of PMTC, the polyester was degraded under alkaline, accelerated conditions. Following the protocol from Nicolas et al., 0.23 g of PMTC-10-8 was dissolved in 9.2 mL THF. [3] A mixture of 0.5 g NaOH in 4.8 mL Methanol was added and the reaction mixture was stirred at 25 °C. The previous colourless reaction mixture turned turbulent, when the degradation products were formed. After 24 h, the reaction mixture was neutralized by slowly adding H_2SO_4. The sample was dried overnight in a vacuum drying oven and analysed by ^{1}H NMR. The degradation product was then used for the biocompatibility tests.

^{1}H-NMR (500 MHz, CDCl3, ppm: H-1 (3.82 ppm, t, 2H, CH_2-OH), H-2 (3.75 ppm, t, 2H, CH_2), H-3 (3.65 ppm, m, 2 H, O-CH_2), H-4 (1.90 ppm, m, 2 H, CH_2-CH_2), H-5 (2.40 ppm, t, 2 H, CH_2-COOH).

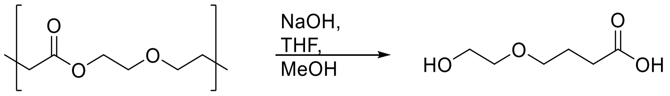

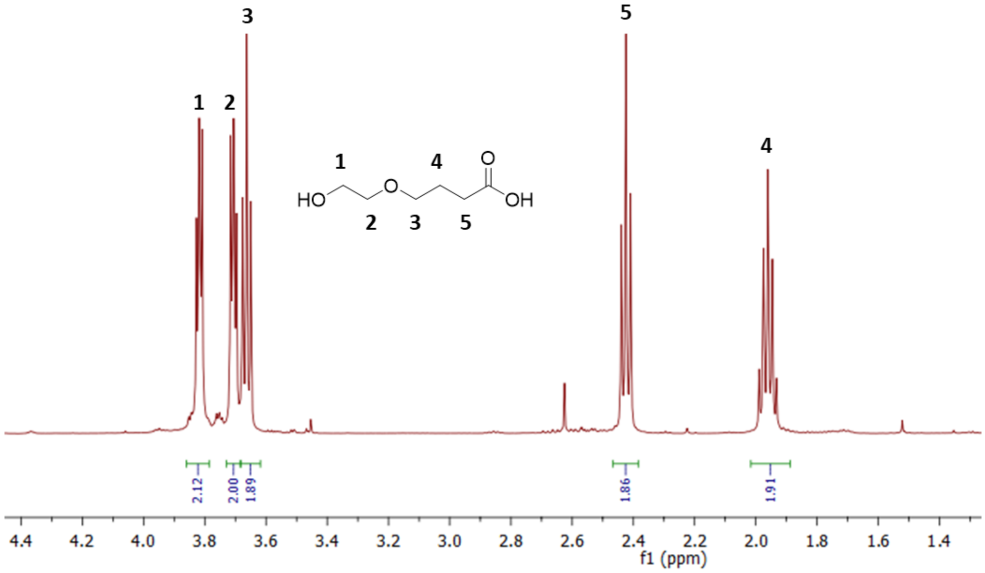

Figure S 1: ^{1}H NMR of the degradation product of PMTC prepared by hydrolysis under accelerated conditions.

1.4. Contact angle measurement

In order to investigate the contact angle, advancing and receding contact angle measurements were performed. The contact line of the drop was forced to advance and recede at a low speed by changing its volume using a OCA15 from DataPhysics. In this limit, the measured values correspond to the static advancing and receding contact angles. To characterize the polymers with as little additional artefacts as possible, smooth films of the polymers were spincoated on silicon wafers. Measuring the receding contact angle, information of the wetted surface are achieved, also allowing insights in polymer-water-interactions.

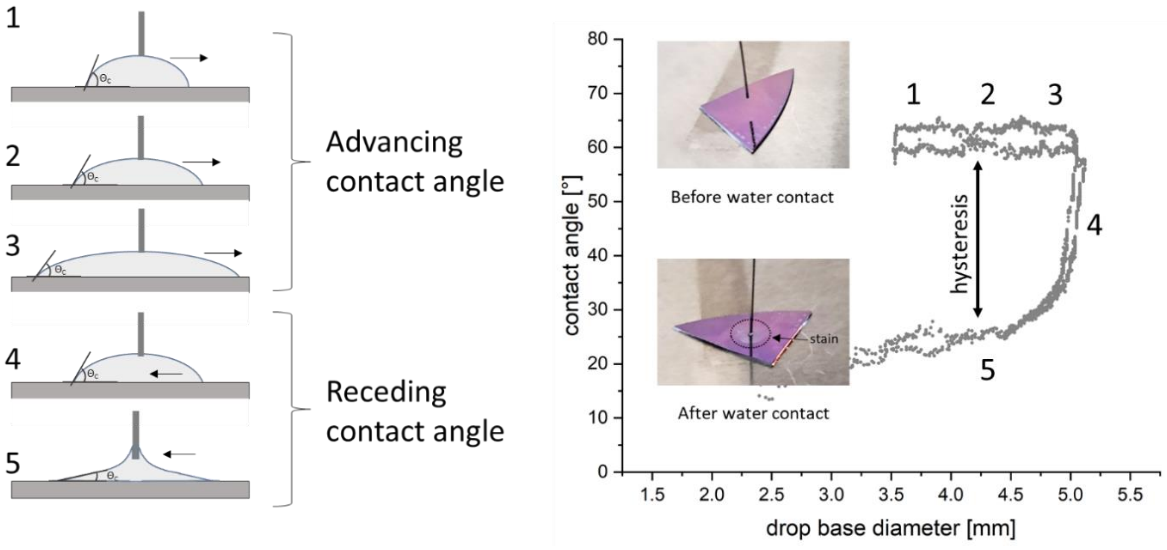

Figure S 2: Evolution of the contact angle left: as a scheme starting with the addition of water (1-3) followed by the removal of the water (4-5). The respective domains and hysteresis is illustrated on the right site in the plot of contact angle vs drop base diameter. The stains obtained from the water-polymer interaction is illustrated in the pictures in the graph.

First, a droplet of water is placed on the surface of the wafer, before a needle connected to a syringe pump is used to slowly add water. With that, the contact line is advancing, more surface is wetted by the water and the overall volume of the droplet is increased (Figure S2, 1-3). The contact line of water, air and the polymer substrate moves slowly away from the centre of the water droplet. In the next step, water is removed and the contact line recedes towards the centre of the water droplet. The drop shrinks (Figure S2, 4-5). Using a camera and image analysis software from DataPhysics (SCA202 V. 4.1.13), the contact angle and drop base diameter were determined.

After preliminary test of the contact angle measurements revealed a staining of the polymer film at the position, where the water droplet was placed, the surface was rinsed with deionized water as an additional cleaning step. After ringing the substrate, it was dipped (3 times) in a beaker of deionized water to remove potentially swollen polymer layers and dust. The cleaned sample was then compared to a sample, which was not cleaned, but only spincoated and dried in a vacuum oven.

Table S 2: Compilation of the results of contact angle measurements of PMTC-9-7 films with different washing techniques or without washing.

	Advancing contact angle Measurement 1	Advancing contact angle Measurement 2
Non-rinsed	64 ± 1 °	59 ± 1 °
Rinsed	54 ± 1 °	56 ± 1 °
Average	58 ± 4 °	

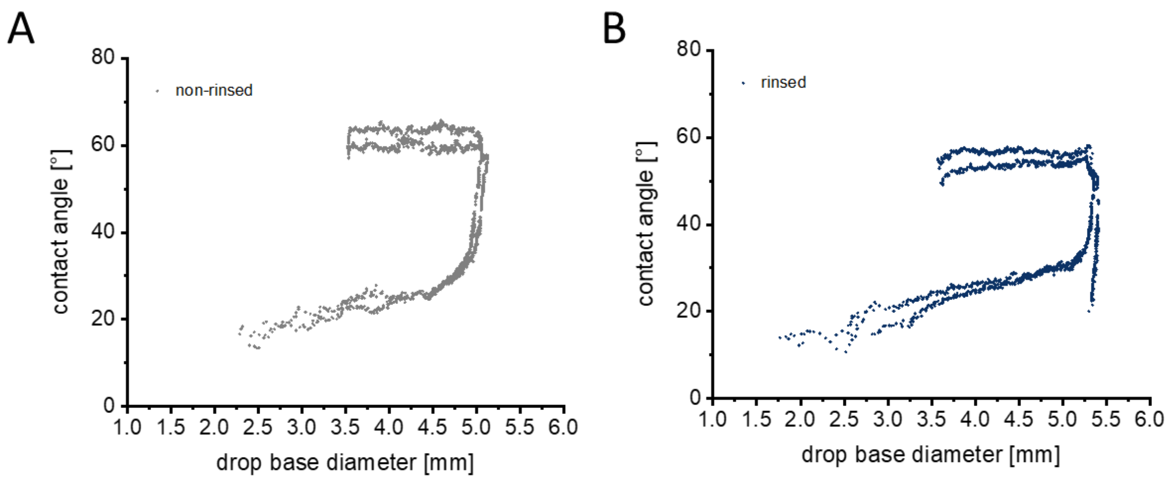

Figure S 3: Compilation of contact angle measurements for PMTC-films A) non-rinsed, B) rinsed with deionized water.

It turned out that the additional cleaning step helped to reduce the contact angle slightly and lead to a slightly lower contact angle. However, the difference of cleaning was a minor effect and the measurement of the contact angle could be validated to be trustworthy. Whilst for these tests, a minimum of 2 different positions at the same substrate were tested, a greater number of samples had to be analysed to allow for better statistics. Since the purpose of the contact angle measurement in this work was to roughly quantify the hydrophilicity of PMTC, this detailed optimization of contact angle measurement should be focus of future work.

2. NPs formation and characterisation

2.1. NP filtration tests

Table S 3: DLS data of the different PMTC-NPs before and after filtration.

Polymer sample	Z-average (pre-filtration)	PDI (pre-filtration)	Z-average (after filtration)	PDI (after filtration)
**PMTC-27-17**	222.3 ± 0.9	0.13 ± 0.02	100.7 ± 2.3	0.11 ± 0.01
**PMTC-9-8**	247.9 ± 4.4	0.13 ± 0.02	128.5 ± 3.5	0.10 ± 0.06
**PMTC-10-4**	288.8 ± 7.7	0.13 ± 0.02	188.6 ± 9.5	0.24 ± 0.33

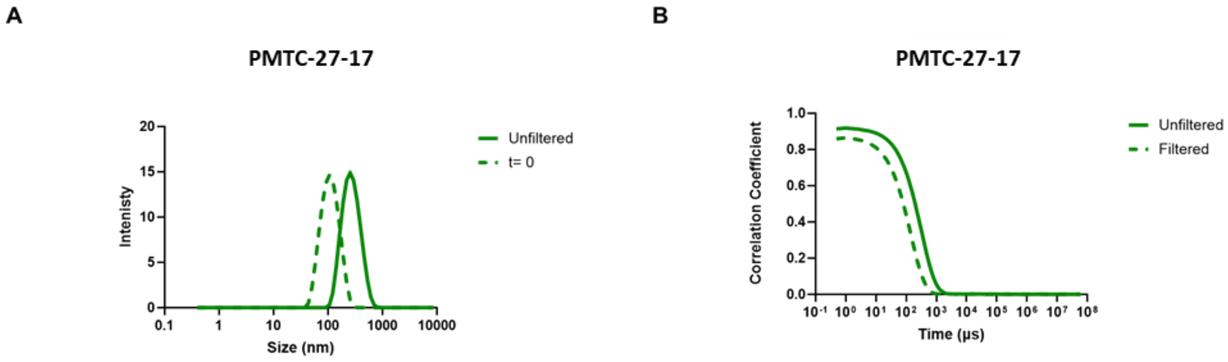

Figure S 4: DLS-traces for a filtered vs unfiltered polymer sample. Same trend for the rest. A. Intensity results. B. Raw Correlation Data.

Filtering is a common technique for sterilising samples for biological testing. Whilst the filtration helped to remove larger particles and dust, the overall Z-average before and after filtration was comparable. A minor difference in the formed nanoparticles' size was observed, but all of the results were within experimental error. Since the filtration did not impact the nanoparticle quality (as seen in the intensity, size distribution and correlation function) and even decreased the PDI of the samples, it was concluded that all NPs would be filtered, as typically done.

2.2. Stability of NPs

Table S 4: DLS data of the different PMTC-NPs stored in deionized water after 0, 7, 14 and 21 days.

Polymer	Z-Average [nm]				PDI			
	t_0	7 d	14 d	21 d	t_0	7 d	14 d	21 d
PMTC-27-17	100.7 ± 2.3	141.2 ± 1.1	138.7 ± 2.2	145.2 ± 2.5	0.11 ± 0.01	0.06 ± 0.02	0.08 ± 0.03	0.09 ± 0.02
PMTC-9-8	128.5 ± 3.5	156.6 ± 0.7	152.0 ± 5.6	160.2 ± 4.0	0.10 ± 0.06	0.09 ± 0.01	0.39 ± 0.08	0.10 ± 0.01
PMTC-10-4	188.6 ± 9.5	185.2 ± 8.9	180.9 ± 8.5	189.9 ± 6.8	0.24 ± 0.33	0.24 ± 0.24	0.10 ± 0.02	0.08 ± 0.03

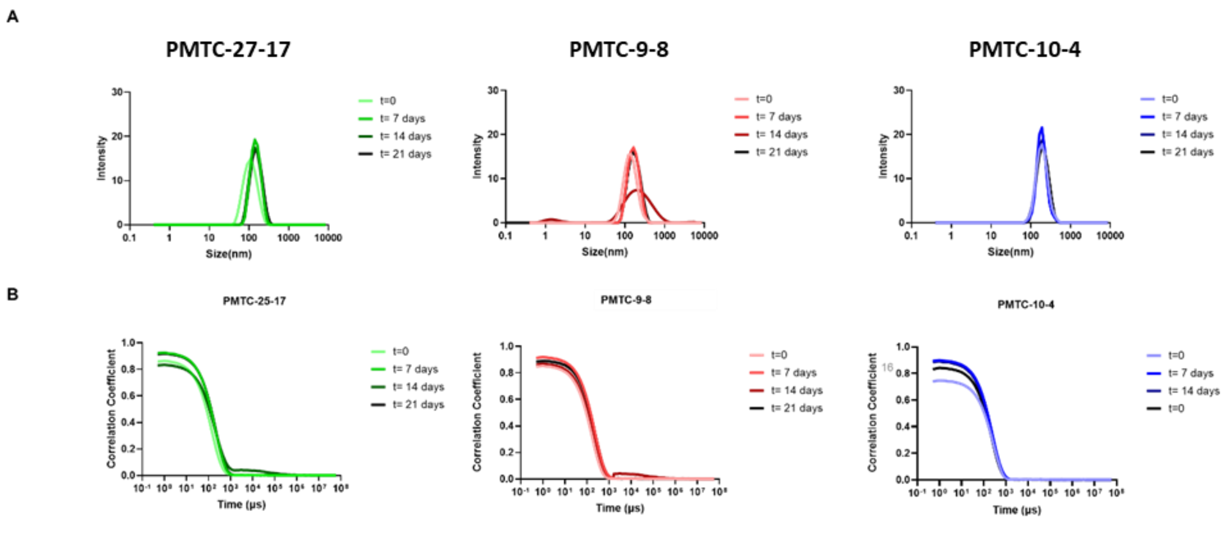

Figure S 5: DLS-traces of the stability test of the polymer samples. A. By Intensity. B. By Correlation Data.

2.3. Stability in different environments

Stability in FBS serum was tested for 24h.

Table S 5: Correlation data of the stability test of the polymer sample for 10% FBS:

Polymer	Z-Average [nm]				PDI			
	DI water	t_0	1 h	24 h	DI water	t_0	1 h	24 h
PMTC-36-17	313.1 ± 34.9	266.6 ± 32.4	170.9 ± 90.1	74.3 ± 15.1	0.37 ± 0.04	0.37 ± 0.02	0.76 ± 0.36	0.92 ± 0.13
PMTC-9-8	358.6 ± 7.6	350.8 ± 12.9	507.7 ± 42.18	246.2 ± 7.6	0.56 ± 0.07	0.53 ± 0.01	0.87 ± 0.16	0.43 ± 0.08
PMTC-10-4	282 ± 9.7	414 ± 9.13	396.9 ± 22.7	327.1 ± 10.7	0.42 ± 0.02	0.71 ± 0.27	0.71 ± 0.29	0.64 ± 0.22

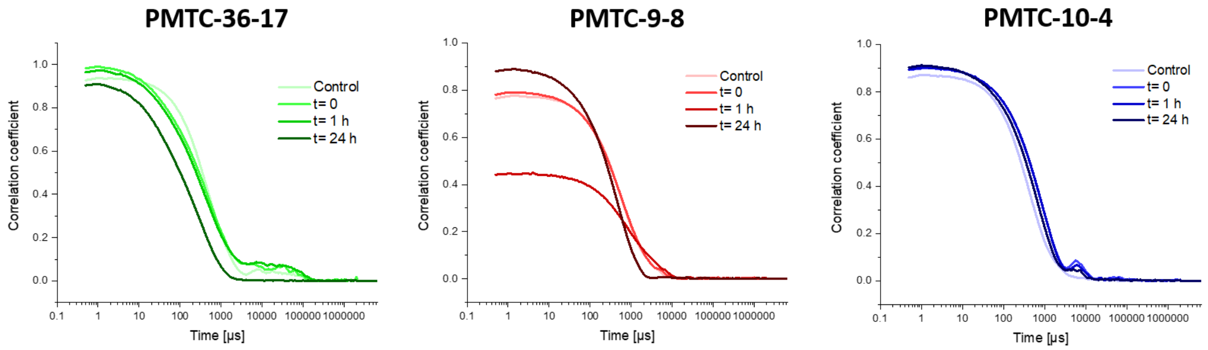

Figure S 6: Correlation data of the stability test of the polymer sample for 10% FBS.

The stability at pH 4 with a citrate buffer was tested for 24 h.

Table S 6: Correlation data of the stability test of the polymer sample for the citrate buffer.

Polymer	Z-Average [nm]				PDI			
	t_0	30 min	3 h	24 h	t_0	30 min	3 h	24 h
PMTC-27-17	221.0 ± 18.3	537.8 ± 100.1	648 .8 ± 91.9	1276 ± 432.3	0.14 ± 0.03	0.53 ± 0.48	0.41 ± 0.52	0.78 ± 0.39
PMTC-9-8	207.9 ± 16.6	352.8 ± 30.2	296.5 ± 14.8	611.6 ± 39.0	0.17 ± 0.03	0.47 ± 0.49	0.08 ± 0.05	0.30 ± 0.15
PMTC-10-4	200.8 ± 14.1	307.7 ± 7.5	656.6 ± 32.9	541.9 ± 27.6	0.16 ± 0.02	0.08 ± 0.06	0.68 ± 0.51	0.17 ±0.06

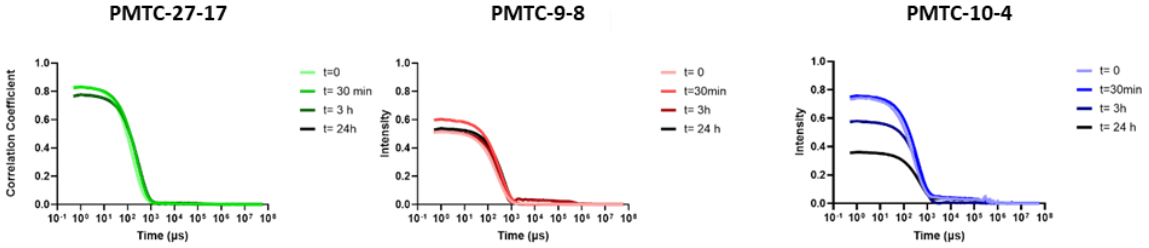

Figure S 7: Correlation data of the stability test of the polymer sample for the citrate buffer.

The stability at pH 7.4 with a PBS buffer was tested for 24 h.

Table S 7: Correlation data of the stability test of the polymer sample for PBS.

Polymer	Z-Average [nm]				PDI			
	t_0	30 min	3 hr	24 hr	t_0	30 min	3 hr	24 hr
PMTC-27-17	121.2 ± 4.7	169.5 ± 7.8	201.6 ± 3.5	197.3 ± 7.3	0.17 ± 0.00	0.17 ± 0.03	0.05 ± 0.03	0.03 ± 0.03
PMTC-9-8	176.9 ± 21.5	177.8 ± 0.0	274.2 ± 3.8	302.4 ± 2.2	0.17 ± 0.11	0.20 ± 0.00	0.73 ± 0.38	0.27 ± 0.13
PMTC-10-4	195.7 ± 3.4	219.2 ± 12.7	329.5 ± 13.9	318.9 ± 8.2	0.29 ± 0.08	0.27 ± 0.03	0.12 ± 0.06	0.14 ± 0.18

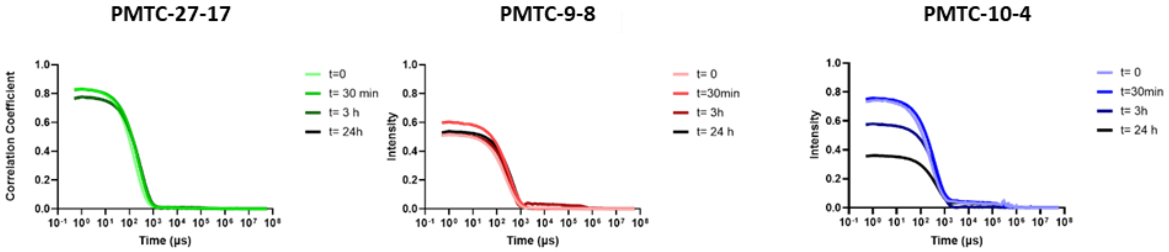

Figure S 8: Correlation data of the stability test of the polymer sample for PBS.

The stability at pH 12 with a sodium phosphate buffer was tested for 24 h.

Table S 8: Correlation data of the stability test of the polymer sample for the sodium phosphate buffer.

Polymer	Z-Average [nm]				PDI			
	t_0	30 min	3 hr	24 hr	t_0	30 min	3 hr	24 hr
PMTC-27-17	204.3 ± 9.7	187.0 ± 8.5	155.4 ± 7.9	242.6 ± 0.7	0.20 ± 0.07	0.17 ± 0.10	0.12 ± 0.02	0.13 ± 0.03
PMTC-9-8	116.6 ± 1.4	124.3 ± 4.5	149.8 ± 1.2	632.8 ± 18.4	0.11 ± 0.01	0.10 ± 0.03	0.36 ± 0.03	0.28 ± 0.21
PMTC-10-4	272.7 ± 9.3	274.0 ± 33.7	215.8 ± 13.4	459.1 ± 8.9	0.50 ± 0.03	0.50 ± 0.37	0.08 ± 0.00	0.28 ± 0.30

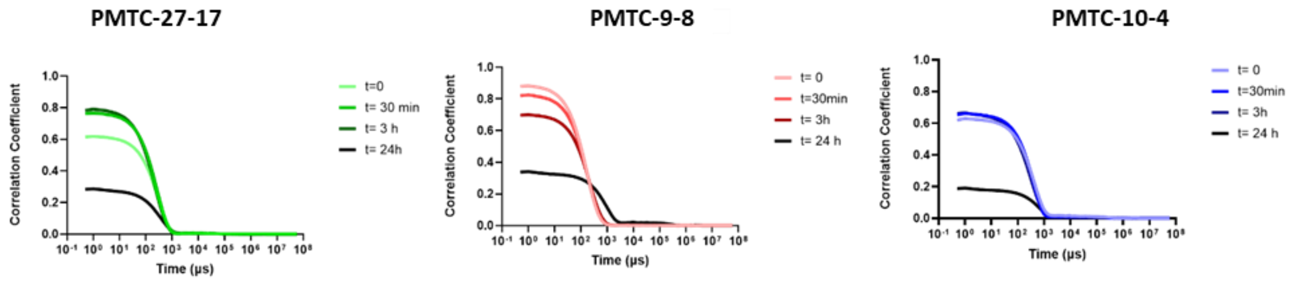

Figure S 9: Correlation data of the stability test of the polymer sample for the sodium phosphate buffer.

3. Enzymatic degradation

DLS results of the enzymatic degradation experiments.

Table S 9: DLS results.

Sample	*Time*	*Z-Average [nm]*	*PDI*
PMTC-27-17	Reference	100.7 ± 2.3	0.11 ± 0.01
	0 min	169.3 ± 21.5	0.22 ± 0.03
	30 min	398.9 ± 10.2	0.44 ± 0.51
	3 h	333.4 ± 0.00	0.54 ± 0.00
	24 h	792.0 ± 0.00	0.53 ± 0.00
	48 h	792.3 ± 17.2	0.30 ± 0.01
PMTC-9-8	Reference	128.5 ± 3.5	0.10 ± 0.06
	0 min	237.3 ± 2.7	0.43 ± 0.50
	30 min	328.1 ± 6.7	0.43 ± 0.506
	3 h	489.4 ± 0.00	0.86 ± 0.00
	24 h	3975 ± 164.0	1.0 ± 0.00
	48 h	1082 ± 30.4	0.80 ± 0.05
PMTC-10-4	Reference	188.6 ± 9.5	0.24 ± 0.33
	0 min	170.1 ± 5.8	0.21 ± 0.12
	30 min	396.5 ± 6.9	0.64 ± 0.51
	3 h	424.6 ± 80.61	0.61 ± 0.56
	24 h	963.2 ± 0.0	0.66 ± 0.00
	48 h	1008 ± 21.9	0.20 ± 0.09

In the following, the DLS-traces as a fundament of Figure 5 was shown.

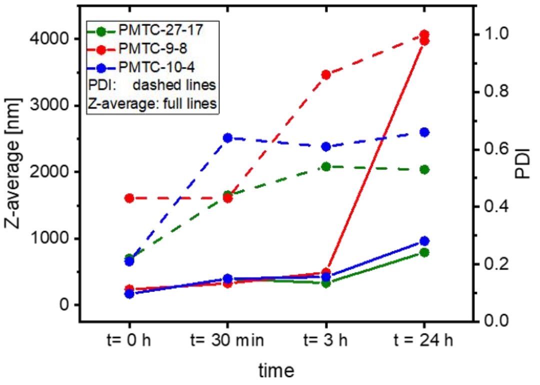

Figure S 10: DLS-results of the degradation studies.

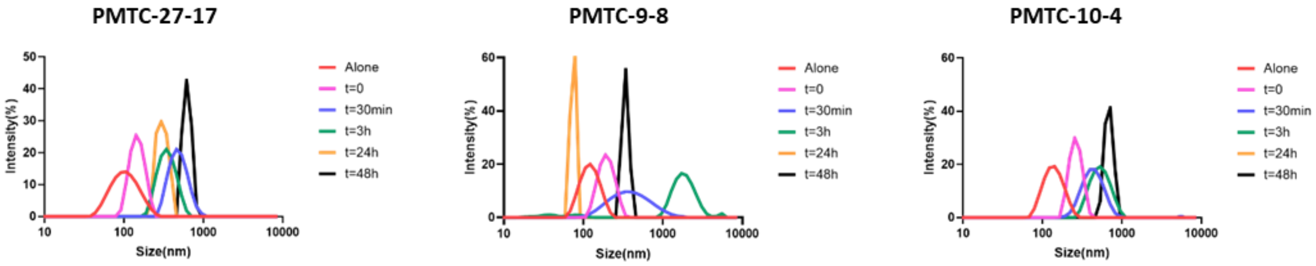

Figure S 11: DLS signals of the enzymatic degradation of PMTC-nanoparticles.

4. Drug encapsulation

Aim of the study was the development of a fully degradable nanoparticle system for drug delivery. In order to investigate, which polymers were most suitable for the encapsulation of a hydrophobic drug, in the next step. Encapsulation studies with Coumarin were performed.

Table S 10: Compilation of DLS data for the different polymers with and without encapsulated Coumarin 6.

Polymer	Z-Average [nm]		PDI	
	Free	Coumarin 6	Free	Coumarin 6
PMTC-27-17	100.7 ± 2.3	117.5 ± 3.5	0.11 ± 0.01	0.15 ± 0.03
PMTC-9-8	128.5 ± 3.5	152.3 ± 1.1	0.10 ± 0.06	0.12 ± 0.09
PMTC-10-4	188.6 ± 9.5	166.0 ± 4.7	0.24 ± 0.33	0.17 ± 0.04

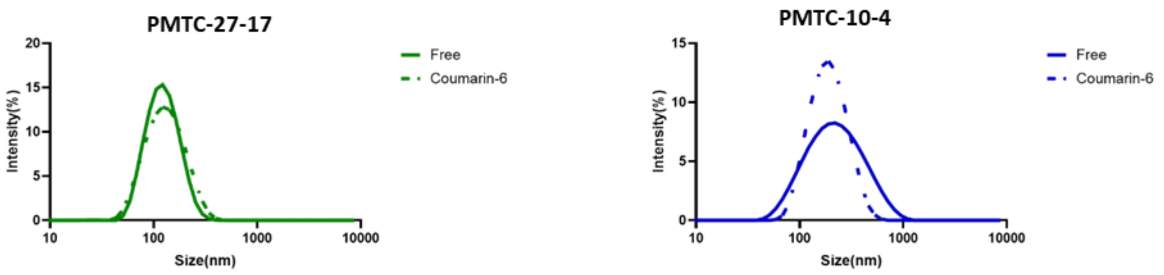

Figure S 12: DLS-traces for free polymers vs encapsulated polymers for the rest of the polymers, PMTC-27-17 and PMTC-10-4.

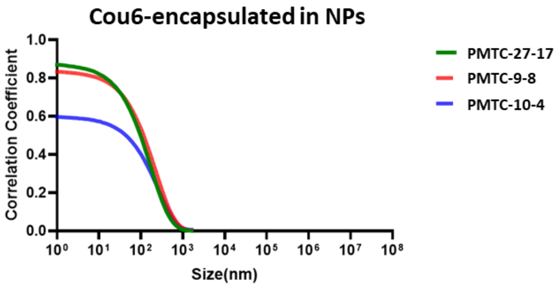

Figure S 13: DLS-traces for the correlation data of the encapsulated polymers.

5. TEM-measurements

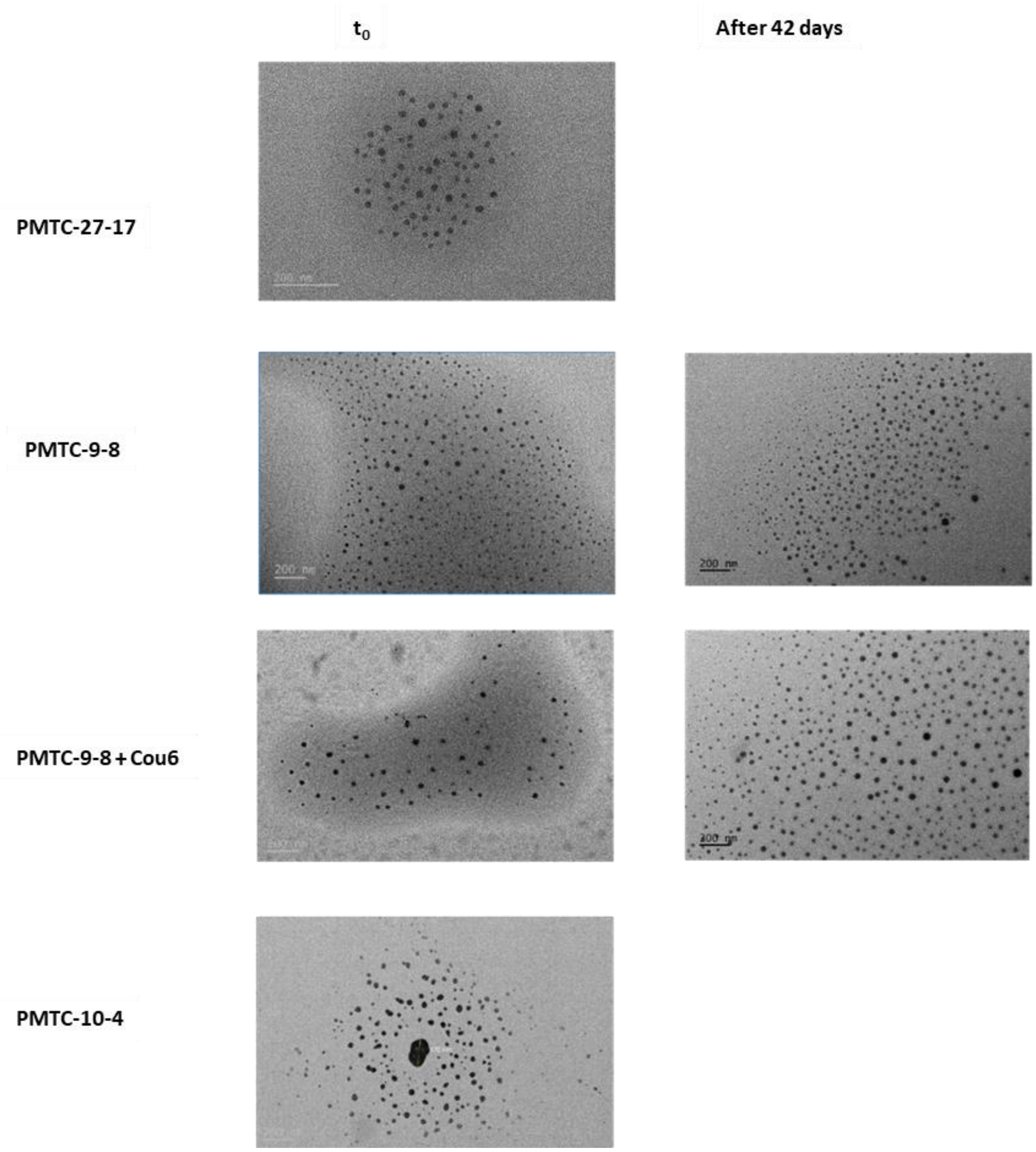

Figure S 14 TEM-image using FEI Tecnai G2 12 Biotwin to allow for visualisation of the prepared NPs

As observed in the TEM images, for all samples spherical nanoparticles were found. However, most of these particles were considerably smaller than the TEM-measurements, with sizes in the sub-40 nm range. Considering the sample PMTC-10/5, a large particle can be seen surrounded by much smaller particles. It can be hypothesised that during the drying process the particles disassemble and shrink in size. Since the particles formed are not self-assembled, but rather colloidally stabilised, water may have served as the stabiliser. Once dried, the forces stabilizing the polymer nanoparticles might decrease trigger a disassembly into smaller polymer particles. However, no traces of small particles were found in the DLS and a limited number of polymer particles with comparable size to the DLS-

results were obtained, supporting this hypothesis of disassembly which was observed for all sample series.

It can be noted from the $t_{42\ day}$ TEM images that most of this disassembly must happen immediately upon dehydration, as no further decrease in size was observed compared to t_0. Conversely, a slight increase in size was observed, perhaps indicating agglomeration of these smaller particles over time.

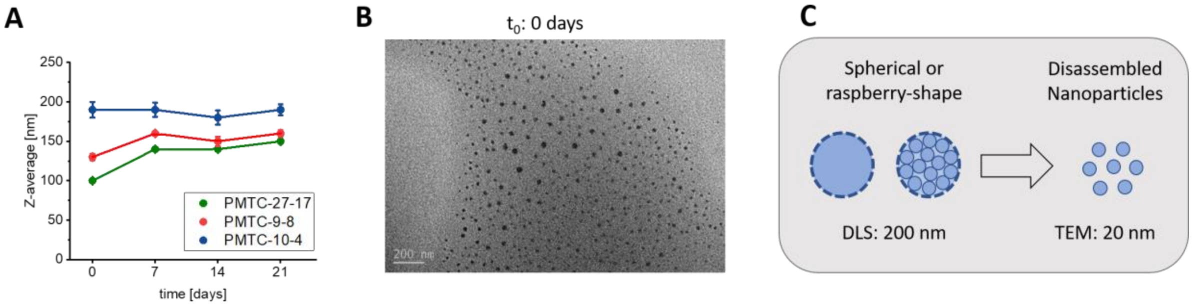

Figure S 15: Results from the size-analysis of the PMTC-based nanocarriers: A) DLS-measurement suggesting a size of 150-200 nm, B) TEM suggesting a size of 20 nm, C) proposed mechanism of a disassembly-process explaining the discrepancy between the two size-determination techniques.

Following the discrepancy between the DLS and TEM-results (Figure S9A and S9B), we propose the following explanation, that could not be validated by additional studies: the DLS-measurements detect the NPs formed in the formulation process. However, due to the low stability of the particles, they appear much smaller on the TEM-grit. In TEM and cryo-TEM. This effect could have three explanations: (i) the shape of the NPs in DLS is an agglomeration of the small NPs observed in the TEM following a raspberry-like-structure, disassembling due to the low forces of interaction with each other (ii) a spherical shape of the particles as a result of swollen particles, that shrink upon drying in the sample-preparation step, or (iii) a disassembly of the particles upon interaction with the TEM-grit due to the low T_g and interactive forces within the prepared NPs, that became apparent only upon sample preparation for the TEM. A proposed mechanism is depicted in Figure S9C. However, following hypothesis (i), the PDI of the prepared particles was surprisingly low, which was not reported for these nanoparticles before. Besides, cryo-TEM would typically allow to freeze the structures in place, which could not be observed. For hypothesis (ii), the same reasoning would apply, but no stable structures could be observed. However, the successful encapsulation of Cou6 observed in the fluorescence-based degradation tests and the comparison of the relative increase in fluorescence activity highlight the encapsulation-potential and support the DLS-measurement and the presence of the reported particles (both spherical or raspberry-like). Whilst the observed increase in size of the NPs within the first days, reported throughout the short- and long-term stability studies and the staining of the polymer film observed in the contact angle-measurements support the swelling of the NPs, this could not be further validated. However, the same reasoning for

the challenging cryo-TEM applies and highlights the challenging analysis of PCKA-based NPs. Following hypothesis (iii), the low interaction forces and the widely discussed low T_g of PMTC reducing mechanical stability of the NPs, in combination with less polymer-polymer interaction upon swelling, this appeared to be the most convincing explanation of the difficult analysis of the presented nanocarrier-system.

Unfortunately, literature never applied PCKA-homopolymers for the formulation of nanoparticles. Whilst various reports on PCKA-based copolymers were established and allowed for well-defined structures, that could be proven by TEM-analysis, to the best of our knowledge, the amount of CKAs never exceeded 50 %. To give a reference for the homopolymerisation of PMTC in a heterogeneous phase in supercritical CO_2, on a macroscopic scale a similar effect of a reduced particle-stability of MDO-N-vinyl-2-pyrrolidone copolymers was also observed, as soon as the amount of MDO increased above a critical value of 20 %, likely due to the reduced T_g.[4] This effect also impacted the polymerisation mechanism as proposed in an earlier study by our group.[5] However, with missing literature references of PCKAs and suitable analytical tools at hand, the details of the behaviour remains unknown and should be focus of future research.

6. References

1. Folini, Jenny; Murad, Wigdan; Mehner, Fabian; Meier, Wolfgang; Gaitzsch, Jens, Updating radical ring-opening polymerisation of cyclic ketene acetals from synthesis to degradation. *European Polymer Journal* **2020,** *134*.
2. Mehner, Fabian; Meissner, Till; Seifert, Alissa; Lederer, Albena; Gaitzsch, Jens, Kinetic studies on the radical ring-opening polymerization of 2-methylene-1,3,6-trioxocane. *Journal of Polymer Science* **2023,** *61* (16), 1882-1892.
3. Théo Pesenti, Julien Nicolas, 100 th Anniversary of Macromolecular Science Viewpoint: Degradable Polymers from Radical Ring-Opening Polymerization (rROP): Latest Advances, New Directions and Ongoing Challenges. *Macro. Lett.* **2020,** (12), 1812-1835.
4. Kwon, Soyoung; Lee, Kyoungwon; Bae, Won; Kim, Hwayong, Precipitation Polymerization of 2-Methylene-1,3-dioxepane in Supercritical Carbon Dioxide. *Polymer Journal* **2008,** *40* (4), 332-338.
5. Mehner, Fabian; Hopkins, Bradley; Reynolds-Green, Morgan; Keddie, Daniel J.; Howdle, Steven M.; Gaitzsch, Jens, Supercritical RROP: Exploring the radical ring-opening polymerisation of 2-methylene-1,3,6-trioxocane in supercritical CO2 as a green solvent. *Polymer* **2024,** *309*.
